# Heading and Then Saccades Predict Visual Discrimination Decisions in Freely Moving Ferrets

**DOI:** 10.1523/ENEURO.0124-26.2026

**Published:** 2026-05-20

**Authors:** Silei Zhu, Tingan Zhu, Abdelrahman Sharafeldin, Marc Mancarella, Farran Briggs

**Affiliations:** ^1^Neuroscience Graduate Program, University of Rochester, Rochester, New York 14642; ^2^Ernest J. Del Monte Institute for Neuroscience, University of Rochester, Rochester, New York 14642; ^3^Center for Visual Science, University of Rochester, Rochester, New York 14627; ^4^Department of Neuroscience, University of Rochester, Rochester, New York 14642; ^5^Neurobiology and Behavior Graduate Program, Columbia University, New York, New York 10027; ^6^School of Mathematics, Georgia Institute of Technology, Atlanta, Georgia 30332; ^7^Brain and Cognitive Science, University of Rochester, Rochester, New York 14627; ^8^Laboratory of Sensorimotor Research, National Eye Institute, Bethesda, Maryland 20892

**Keywords:** decision-making, eye tracking, ferret, freely moving, head tracking, saccade, visual discrimination

## Abstract

Decision-making is a continuous process that manifests as evolving sequences of motor movements while animals navigate the sensory environment. Studying decision-making in a naturalistic setting has been challenging as restrictions are typically imposed on subjects’ motor actions in the laboratory. We utilized a novel paradigm in which animals move freely throughout the decision-making process to examine the sequence and timing of motor actions predictive of decisions. We trained freely moving ferrets (two males, three females), highly visual carnivores, to perform visual discrimination tasks and measured their head position and eye movements to assess the temporal dynamics of heading and saccades during visually guided decisions. We discovered that heading revealed ferrets' “turning time” per trial, signaling their choices, and heading on its own best predicted ferrets' decisions. Ferrets made decisions quickly and decisively, although total trial durations varied across animals. Importantly, initial heading, at the beginning of the decision-making process, revealed ferrets' decision biases and task strategies. Ferrets made choice-directed saccades on most trials. Horizontal eye movements and saccades were also predictive of decisions, but saccades followed choice-indicative head turns. These results show that ferrets make quick decisions with minimal visual scanning and then orient first with their heads and then with saccades toward targets displaced by >10°. Furthermore, our findings indicate that heading is the most robust predictor of visually guided decision-making, followed by saccades. Together, these motor actions provide reliable, noninvasive readouts of the temporal dynamics of natural visual decision-making in freely moving subjects.

## Significance Statement

A fundamental challenge in neuroscience is to understand how cognitive processes like making decisions occur during natural behaviors. Recent technological advances have made it possible to rigorously measure body and eye movements in freely moving animal subjects. Here, we leverage these capabilities to determine whether heading or eye movements predict decisions in ferrets performing visual discrimination tasks. We show that ferrets indicate their choices by orienting first with their heads and then with their eyes. Heading position also revealed animals' individual biases. These results demonstrate that heading is the most reliable indicator of individual subjects' decision-making in real time, providing a noninvasive readout of cognition in freely moving subjects.

## Introduction

A major goal in biology is to understand how organisms make decisions and execute behavioral actions. Motor actions like eye movements have been utilized as behavioral readouts of decisions across a variety of species (Gallivan et al., 2018). However, the precise temporal relationships between motor actions and cognitive decisions are not known. Furthermore, decision-making is typically measured in a laboratory setting in which subjects' motor actions are restricted. To better approximate a naturalistic decision-making process, we observed decision-making in freely moving animals. We selected ferrets, diurnal carnivores with good vision (Jackson and Hickey, 1985), as our animal model to probe the continuous process of decision-making through head and eye tracking in ferrets performing visual discrimination tasks.

In natural visual behaviors, animals orient their eyes and head toward objects of interest. Multiple groups have recently demonstrated novel methods for eye and head tracking in freely moving primates, carnivores, and rodents (Shepherd and Platt, 2006; Singh et al., 2025; Wallace et al., 2025), with some studies showing accompanying changes in neuronal activity during movement (Milton et al., 2020; Mao et al., 2021; Parker et al., 2022). Consensus across primate and carnivore studies is head and eye movements are coordinated by common commands in a flexible manner, dependent on the amount of gaze reorienting required (Bizzi et al., 1972; Guitton et al., 1990; Galiana and Guitton, 1992; Freedman, 2008), perhaps with the goal of bringing relevant targets onto the region of the retina with highest cone density (Wallace et al., 2025). In humans and nonhuman primates (NHPs), the timing and amplitude of head movements relative to voluntary, ballistic eye movements called saccades, depend on the amount of desired gaze shift: larger shifts involve head movements proceeded by saccades while smaller shifts involve smaller amplitude saccades followed by head movements (Zangemeister and Stark, 1982; Freedman and Sparks, 1997). Head and eye movement coordination in cats is similar (Galiana and Guitton, 1992); head movements in cats can precede saccades by 30–40 ms (Blakemore and Donaghy, 1980; Guitton et al., 1984). In rodents, orienting may be guided by head movements (Skyberg and Niell, 2024) followed by saccade-like eye movements to recenter the eye (Meyer et al., 2020; Michaiel et al., 2020). Accordingly, head movements explain a large fraction of eye position variability in rodents (Wallace et al., 2013; Meyer et al., 2018) and reflect head movements they cannot make when head-fixed (Meyer et al., 2020). Given recent technological innovations enabling head and eye tracking in freely moving animals, we asked: how and when do head and eye movements coemerge and contribute to decision-making throughout the active decision process?

Coordination of head and eye movements to acquire visual targets in non-head-restrained animals is well established (Freedman, 2008), but studying these movements during active decision-making is not. Visual decision-making is typically studied in humans and NHPs performing visual tasks, while their heads are stabilized and unmoving (Schall, 2001; Sugrue et al., 2005; Gold and Shadlen, 2007). Visual decisions are often reported by saccades using eye tracking cameras or scleral coils (in NHPs; Kimmel et al., 2012). Saccade metrics—velocity, latency, position error, amplitude, and pupil size—inform how visual stimulus evidence is accumulated and decisions made (Spering, 2022). To examine how unrestrained and more natural eye and head movements coordinate to facilitate visual decision-making, we studied ferrets due to their higher visual acuity and larger binocular fields (Law et al., 1988) relative to rodents (Drager, 1974; Prusky and Alam, 2013; Leinonen and Tanila, 2018). Ferrets can perform more challenging visual discrimination tasks (Pollard et al., 1967; von Melchner et al., 2000; Hupfeld et al., 2006; Hupfeld et al., 2007; Hollensteiner et al., 2015; Dunn-Weiss et al., 2019) compared with rodents (Baker, 2013; Leinonen and Tanila, 2018), and they make head-orienting movements during sound localization tasks (Nodal et al., 2008). We utilized a two-alternative forced choice paradigm in which ferrets needed to assess visual stimuli presented on a monitor, orient, and locomote toward the correct reward port displaced ∼30° to the left or right of the monitor center. We synchronously measured heading angle and eye movements to determine how ferrets made visual discrimination decisions. Surprisingly, ferrets made fast and decisive decisions and their horizontal eye movements followed head turning toward reward ports, suggesting that ferrets utilized central and peripheral vision to quickly assess visual stimuli with minimal scanning. Heading was the most robust predictor of visual decision-making and also provided insight into ferrets' biases and strategies.

## Methods and Materials

### Animals

Data for this study were obtained from three adult male ferrets and two adult female ferrets (*Mustela putorius furo*; age range, 1–5 years). All animal procedures were approved by the Institutional Animal Care and Use Committee at the University of Rochester Medical Center and adhered to the guidelines for animal use issued by the US Department of Agriculture and NIH. Ferrets were water-regulated for behavioral training and testing. Ferrets received water rewards and performed tasks until they were satiated during training and testing days, which totaled no >5 d per week.

### Behavioral box

**Figure 1.**
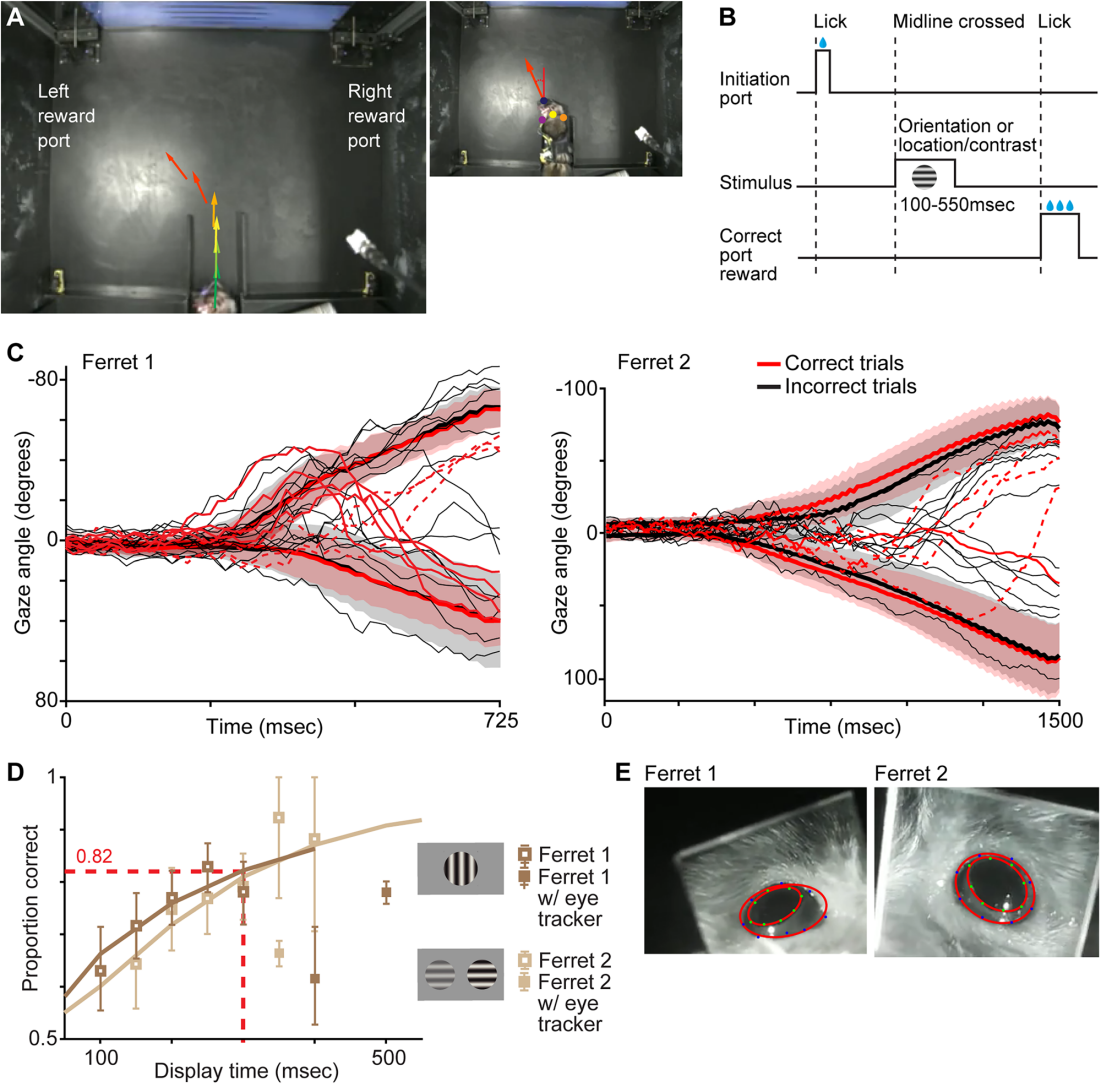
Tracking ferrets' head and eye during visual discrimination tasks. ***A***, Overhead single video frame of Ferret 1 in the behavioral box performing an orientation discrimination trial. This video frame shows Ferret 1 crossing the midline IR beam, which triggered display of an oriented grating on the monitor. Colored arrows indicate tracking of head direction over subsequent frames as the ferret approached the left reward port. Inset at right shows a video frame from a different session in which Ferret 1 is approaching the left reward port. Blue, yellow, orange, and magenta dots are DeepLabCut tracking dots used to estimate heading angle relative to the monitor center (red arrow with dashed angle relative to vertical red line). ***B***, Visual discrimination task structure. Ferrets initiated trials by licking at the initiation port for a small amount of water, then crossed the midline to trigger visual stimulus display, and then moved to the correct reward port based on the orientation or location/contrast of the stimulus to receive a reward. ***C***, Gaze trajectories (heading + horizontal eye position) for Ferrets 1 and 2 from stimulus display onset (time, 0) to twice the time to reach a heading-based choice predictivity AUC of 0.95 per ferret (see Materials and Methods). Thick red and black lines are mean gaze on correct and incorrect trials, respectively, with shaded regions illustrating standard deviations (SDs). Gaze trajectories are plotted separately for left port (upper curves, negative angles relative to monitor center) and right port choices (lower curves, positive angles). Thin red and black lines are single-trial examples, some with wandering behavior; dotted red lines show leftward correct trial examples for clarity. ***D***, Psychometric curves for Ferrets 1 and 2 performing orientation and contrast discrimination tasks, respectively, with varying visual stimulus display duration. Squares are means across sessions; error bars are SEMs. Sessions without (open squares) and with eye tracker (filled squares) are shown. The display duration corresponding to 82% accuracy (red dashed line) was taken as the display duration required for ferrets to complete the task. Both ferrets exhibited reduced performance on varying display duration trials while wearing eye trackers. ***E***, Example video frames of eye tracking per ferret. Boundaries of pupils and eyelids were automatically labeled using DeepLabCut and fitted with ellipses (red). Extended Data Figure 1-1 illustrates distributions of total trial durations per ferret.

We designed a behavioral box (Fig. 1*A*) for freely moving ferrets to perform visual discrimination tasks according to Dunn-Weiss et al. (2019), with some modifications. The standard behavior box measured 2 × 3 ft with a 24 inch LCD monitor (VPixx Technologies) behind museum glass on one side. The box had three water dispensing ports: an initiation port on the wall opposite the monitor and two reward ports positioned left and right of the monitor. Ferrets initiated trials by licking at the initiation port and then indicated their behavioral choice by licking at the left or right reward port. Infrared (IR) beams detected contact with each port by beam break, after which water was dispensed through solenoid valves (Parker Hannifin) for correct choices. A fourth IR beam (midline IR beam), positioned halfway between the initiation port and the monitor, indicated when ferrets crossed the midline and triggered visual stimulus display. Our modification to the standard behavioral box included addition of barriers on each side of the box between the initiation port and the monitor requiring that ferrets cross the midline from the center of the box. These barriers created a corridor perpendicular to the midline so that ferrets faced straight ahead, toward the monitor, while passing through the midline (Fig. 1*A*). The barriers ended at the midline, allowing ferrets to move left or right toward the reward ports after crossing the midline. An Omniplex data acquisition system (Plexon) recorded timestamps for all IR beam breaks. All visual stimuli were generated on the VPixx monitor with PsychToolbox (Brainard, 1997; Pelli, 1997; Kleiner et al., 2007). PLDAPS (Eastman and Huk, 2012) programs supported precise control of stimulus and event timing.

### Visual discrimination task paradigm

All visual discrimination trials followed the same sequence (Fig. 1*B*). Ferrets initiated a trial by activating the initiation port where they received a small amount of water. Then they turned around and crossed the midline IR beam. The break of the midline IR beam triggered visual stimulus display. For most sessions, visual stimulus display duration was fixed at a value between 300 and 500 ms. On a subset of sessions, stimulus display duration varied between 100 and 550 ms. Ferret 1 performed an orientation discrimination task with a single, centrally positioned vertical or horizontal static grating (100% contrast for eye tracking sessions and 51% contrast for other sessions, 0.2 cycle/°, 30° diameter). Importantly, all visual stimuli were placed at a vertical position on the monitor corresponding to ferrets' eye level while standing in the behavioral box, which we refer to from here on as vertical position zero. Degrees of the visual angle (i.e., for stimulus diameter and horizontal position) were based on a viewing distance of 55 cm from the midline of the behavioral box to the monitor. Ferrets 2–5 performed contrast discriminations with a single static grating (17–100% contrast, 0.2 cycle/°, 15° diameter) positioned on the left or the right side of the monitor (vertical position, 0; horizontal position, 15°) or two static gratings (48 and 3% or 45 and 6%, 0.2 cycle/°, 15° diameter) simultaneously presented on both sides of the monitor (vertical, horizontal positions as above). Right and left reward ports were assigned to vertical and horizontal grating orientations, respectively, for the orientation discrimination task. For the contrast discrimination, ferrets needed to select the reward port next to the higher contrast stimulus. The orientation or the location/contrast of the grating(s) was varied using a staircase or pseudorandomized so that every session consisted of an equal number of trials in which the left and right ports were the correct choice and an equal number of trials per contrast level. Ferrets received a large water reward for choosing the correct reward port and no reward for choosing the incorrect port. The trial ended after ferrets chose a left or right port. A new trial was initiated by activating the initiation port. There were no time-outs and voluntary trial initiation resulted in few-to-no aborted trials. Sessions ended when ferrets failed to initiate a trial for over 1 min. Because all sessions utilized the pseudorandomized trial structure, chance performance was 50% for all tasks. The number of total trials completed, overall accuracy (percentage of correct trials relative to total trials), and total trial time were computed per trial per ferret (Table 1). Wilcoxon rank-sum tests were used for comparisons between distributions of accuracy and total trial time with versus without the eye tracker for Ferrets 1 and 2 because these distributions were not Gaussian.

### Adjusting visual stimulus display duration based on performance

As described above, visual stimulus display was triggered by the midline IR beam break. To measure the visual stimulus display duration required for ferrets to perform tasks accurately, on a subset of trials, we varied the visual stimulus display duration between 100 and 550 ms pseudorandomly so that every session consisted of an equal number of trials with each visual stimulus display duration. Behavioral psychometric curves were generated by plotting the proportion of correct choices against visual stimulus display duration (Fig. 1*D*). The visual stimulus display duration corresponding to a proportion correct of 0.82 was taken as the display duration required for consistent task completion. During eye tracking, Ferrets 1 and 2 needed to wear a vest with onboard electronics, which slowed both ferrets down (Table 1, Extended Data Fig. 1-1). We therefore separately measured the required display duration for task completion while Ferrets 1 and 2 wore the eye tracker and vest.

Animals were considered fully trained once they were able to perform a task at a minimum of four different contrast levels or achieved a consistent correct rate >70% (Table 1). Once trained, ferrets performed on average 89–132 correct trials per session/day (Table 1). Post-training sessions with <70 correct trials were excluded from further analysis, except for Ferret 2's sessions with eye tracking in which this animal sometimes performed fewer trials and except for Ferret 5 that performed on average 56 trials per day during sessions in which the visual stimulus display time varied.

### Headpost surgery and maintenance

Surgeries to implant platforms for head-mounted eye trackers were performed for Ferrets 1 and 2 under full surgical anesthesia and using sterile procedures. A midline skin incision was made over the frontal region of the skull and the muscles retracted to create a space for eye tracker platform placement. The skull was etched with enamel etchant; then a custom-made aluminum platform (4.7 × 20 mm) was secured to the skull adjacent to the sagittal crest using C&B Metabond (Parkell) and/or bone cement (Zimmer Biomet). A spacer was screwed to the platform to prevent tissue from growing over it. Ferrets recovered from the surgery for at least 1 week. After that, the spacer and the margins of the platform and skin edge were cleaned with chlorhexidine solution at least twice per week. For eye tracking, the spacer was removed, and the eye tracker was screwed to the platform for testing and then removed, and the spacer was replaced after each recording.

### Head tracking

An overhead camera (Stingray; frame rate, 70 or 80 Hz) connected to a Cineplex data acquisition system (Plexon) was installed above the behavioral box to record ferrets' movements. The Cineplex data acquisition was directly connected to the Omniplex acquisition system recording behavioral events. Timing of the overhead camera (Cineplex-Omniplex), the eye tracking data stream (Raspberry Pi Zero 2 W), and the behavioral data stream (VPixx-Omniplex) were synchronized at the start of each session by a flashed LED digitally triggered by the VPixx PC. The flash was directly recorded in the behavioral data stream (along with IR beam breaks at reward ports and the midline, etc.), by Cineplex-Omniplex and by the Rasberry PI minicomputer. We also incorporated a secondary synchronizing signal by having the VPixx PC also trigger a flash on the eye tracker IR LED at the same time as the external LED. All subsequent time stamps were aligned across data streams to this initializing LED flash.

**Figure 2.**
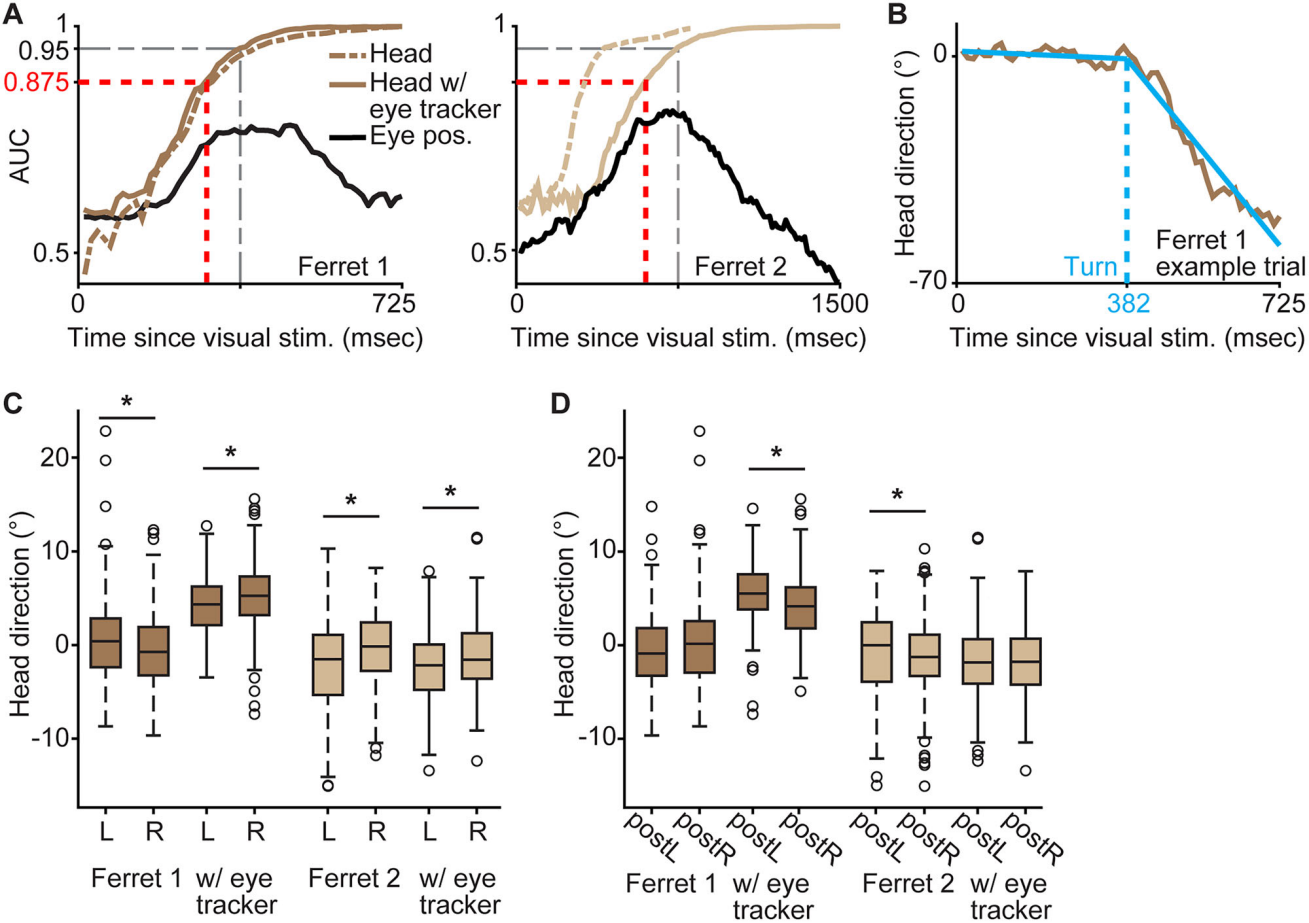
Head tracking indicates decision time, turning time, and decision bias. ***A***, AUC, calculated per video frame across all trials for each ferret, indicates predictivity of head direction without (dashed brown line) and with eye tracker (solid brown line) and eye position (black line) of correct choice. AUC of 0.875 (red dashed lines) is the decision criterion from which decision time was assessed; AUC of 0.95 (gray dashed lines) is shown for comparison. Head and eye analysis durations were set at 2× the time it took for the AUC of head direction to reach 0.95 (unique to each ferret and eye tracking condition; Table 2), so timescales (*x*-axes) are different for Ferrets 1 and 2. ***B***, Head turning time (dashed blue line) was determined per trial as the intersection of a bilinear function (solid blue line) fit to head angles (brown line) following visual stimulus onset. ***C***, Distributions of head direction at stimulus onset prior to left (L) or right (R) choice per ferret without and with eye tracker. Black lines are medians; the bottom and top edges of the boxes indicate the 25th and 75th percentiles, respectively; whiskers indicate the range of data not considered outliers; and circles are outlier data points. Asterisks indicate significant differences between eventual left/right choices (see Tables 3, 4 for statistics). ***D***, Distributions of head direction at stimulus onset following left (L) or right (R) choice on the previous trial per ferret without and with eye tracker. Conventions as in ***C***, see Tables 3 and 4 for statistics. Extended Data Figure 2-1 illustrates decision time, turning time, and decision biases for Ferrets 3–5.

We used DeepLabCut (Mathis et al., 2018; Nath et al., 2019; Lauer et al., 2022) to train an artificial neural network (ANN) to automatically label each ferret's neck and nose, placing one dot on the nose and three dots marking the center and lateral sides of the neck (Fig. 1*A*, inset; Movie 1), after training the ANN with ∼50 video frames in which these body parts were manually labeled. Frames were excluded if the likelihood of the ANN's predictions for the nose or neck markers fell below 0.9. Head direction was calculated per video frame as the angle between a line connecting the neck and the nose and a line connecting the neck and the center of the monitor. The Cineplex system was synchronized with the Omniplex data acquisition system such that timing of heading was relative to midline IR beam breaks and reward port IR beam break time stamps. Wilcoxon rank-sum tests were used for comparisons between distributions of heading angle at visual stimulus onset before left or right choices (Fig. 2*C*; Extended Data Fig. 2-1*B*; Tables 3 and 4) and for comparisons of the heading angle following left or right choice on the previous trial (Fig. 2*D*; Extended Data Fig. 2-1*C*; Tables 3 and 4), because heading angle distributions were not Gaussian after grouping based on the choice in the current or previous trial.

**Movie 1.** Ferret 1 performing several trials of the orientation discrimination task while wearing the eye tracker plus associated accessories. DeepLabCut tracking dots follow the nose (blue) and neck (yellow, middle; orange and magenta, lateral). [View online]

### Eye tracking

We designed a head-mounted eye tracker based on Meyer et al. (2020). The head-mounted eye tracker included an LED that shined IR light into the eye, an IR-reflective mirror that was transparent to visible light and placed in front of the eye, and a camera (Raspberry Pi Camera Module 3; frame rate, 80 Hz) recording eye movements from the mirror. The eye tracker was assembled from 3D-printed parts which were customized for each ferret to optimize the camera view of the pupil and the eyelids. The head-mounted parts for each eye tracker weighed 21 g. Ferrets wore a vest to carry a minicomputer (Raspberry Pi Zero 2 W) that controlled video recording and powered the IR LED, as well as a battery that powered the minicomputer. The weight of the vest plus minicomputer and battery was 76 g. We tracked Ferret 1's left eye and Ferret 2's right eye (Fig. 1*E*), under the assumption that left and right eye movements were conjugated (Wallace et al., 2025). The contours of the pupil and the eyelids of each animal were automatically tracked using a different DeepLabCut-based ANN trained with ∼50 video frames in which pupil and eyelids were manually labeled by fitting with ellipses (Fig. 1*E*; Movie 2). Frames were excluded if the likelihood of the ANN's predictions for any pupil or eyelid marker fell below 0.9. The diameter of the eyeball was approximated by the major axis of the eye ellipse. The center of the eye ellipse was set as the origin, from which we calculated the angular distance to the center of the pupil ellipse. This eye position estimate may not have reflected true eye-in-head position because (1) precise eye tracker calibration is not possible in a non-head-fixed and non-fixating animal and (2) a slightly offset position of a mirror relative to the optical axis of the eye causes pupil ellipse deformation. Due to these constraints, we only analyzed measures that did not depend on calibration: horizontal direction of eye movements and timing of saccades. Horizontal eye position and head angle were measured synchronously, so, given the fixed position of the camera, we could estimate horizontal eye movements in relation to head rotation. Vertical eye movements were poorly predictive of decisions (data not shown) likely because choice ports (i.e., potential gaze targets) were displaced horizontally but close to vertical position zero.

**Movie 2.** Eye tracking video of Ferret 1 performing several trials of the task. DeepLabCut tracking dots outline the pupil. [View online]

### Estimation of decision time based on head movements

To estimate decision time per ferret and task, irrespective of visual stimulus display duration or presence/absence of the eye tracking apparatus, we quantified the predictivity of head direction for behavioral choice at each Cineplex-recorded video frame (14.2 or 12.5 ms per frame) following the onset of the visual stimulus. We used an analysis of the area under the receiver operating characteristic (ROC) curve to compute heading predictivity. The ROC curve expressed the true positive rate against the false-positive rate of predicting choices from all possible cut points in the distribution of a ferret's head direction (distributions included all trials from all sessions at a given time point following visual stimulus onset per ferret). The area under the ROC curve (AUC) summarized the prediction accuracy across all possible cut points to quantify how well each ferret's head direction at a given time point following visual stimulus onset predicted their subsequent choice. Curves showing AUC values corresponding to each time frame following visual stimulus onset were generated (Fig. 2*A*; Extended Data Fig. 2-1*A*). Confidence intervals for each AUC curve were calculated at the time point corresponding to the visual stimulus display duration required for consistent task completion, estimated from the psychometric curve described above (Fig. 1*D*): for Ferret 1, the confidence interval was [0.85, 0.90], and for Ferret 2, the confidence interval was [0.82. 0.88]. Because 0.875 fell within the confidence intervals for both ferrets and it was also within the commonly accepted criteria for excellent predictivity: 0.8–0.9 (Mandrekar, 2010), we defined AUC ≥ 0.875 as the criterion for ferrets' choice of reward port. The time point of the first video frame after which AUC was above 0.875 for two consecutive video frames was determined to be each ferret's decision time.

### Estimation of head turning time

To measure head turning per trial, we first established a protocol for determining the heading temporal analysis window per ferret, as ferrets moved at different speeds (Table 1, Extended Data Fig. 1-1). Each analysis window started at visual stimulus onset. The duration of each ferrets' analysis window was twice the time to reach a heading-choice predictivity AUC of 0.95 (described above). We next computed heading angle per video frame within this analysis window. We then fit a bilinear function to the heading angle over time curve per trial (Fig. 2*B*). The intersection of the bilinear function was taken as the turning time per trial, i.e., the time the ferret turned its head away from the monitor and toward one of the reward ports. To compare turning time (from all trials per ferret) to that ferret's decision time, a one-sample *t* test was used (Table 2), because the decision time was a single value per ferret/task estimated from the AUC (as described above).

### Estimation of decision based on horizontal eye position

**Figure 3.**
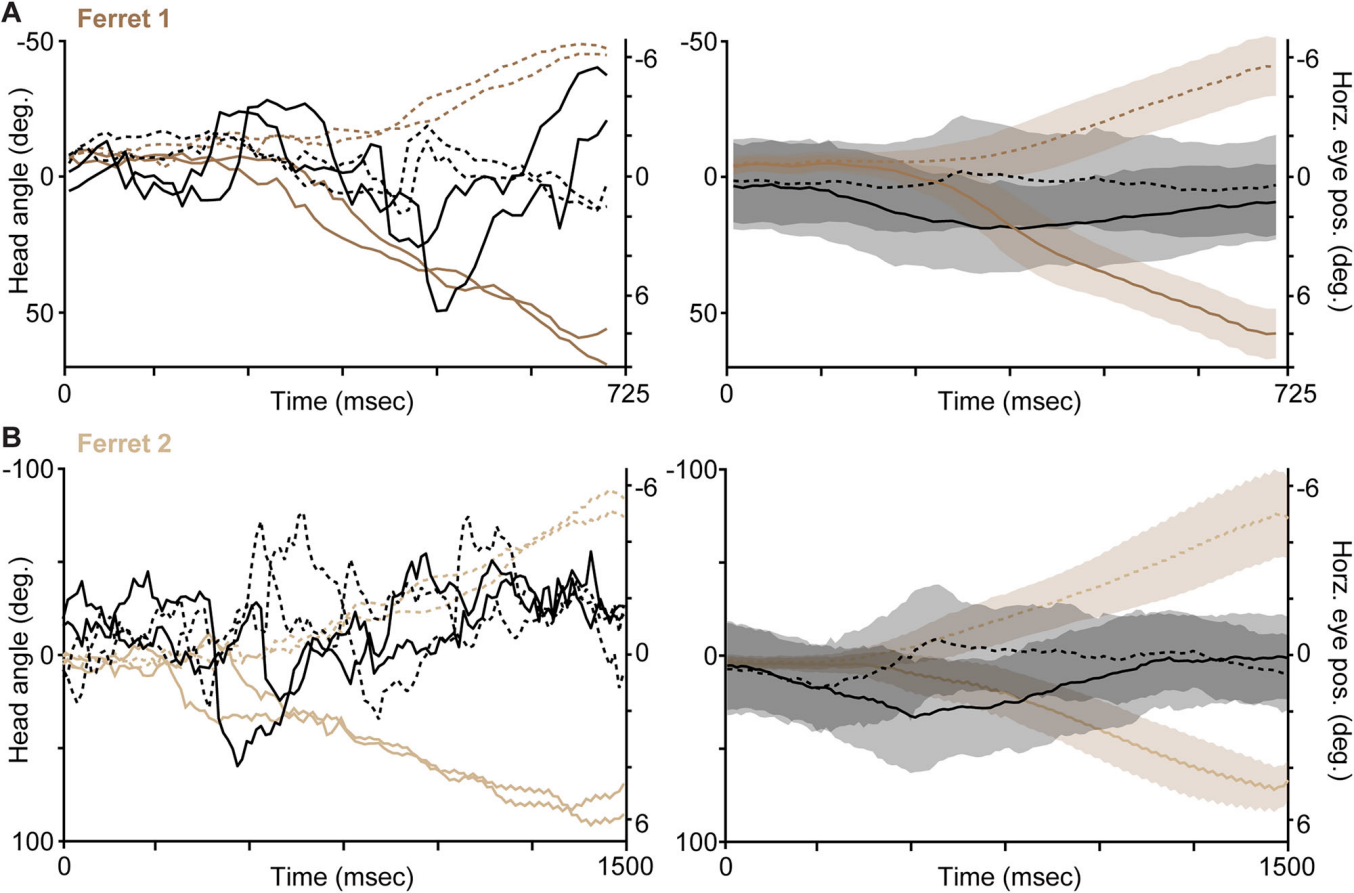
Time-aligned heading and horizontal eye position. ***A***, Time-aligned head angle (brown) and horizontal eye position (black) for Ferret 1 from stimulus display onset (time, 0) to twice the time to reach a heading-based choice predictivity AUC of 0.95 (see Materials and Methods). Left illustrates four example trials; right illustrates mean and SD (shading) head angle and horizontal eye position. Trajectories are plotted separately for left port (upper curves and dotted lines, negative angles relative to monitor center) and right port choices (lower curves, positive angles). Note differences in scale for head angle (left axes) and horizontal eye position (right axes). ***B***, Time-aligned head angle and horizontal position for Ferret 2, conventions as in ***A***. Extended Data Figure 3-1 illustrates average horizontal eye position around turning time per ferret.

Using the same temporal analysis window described above for head turning (unique to each ferret), we also extracted the horizontal eye position per eye tracker video frame (12.5 ms per frame). We computed gaze trajectories per trial as the sum of time-aligned heading and horizontal eye position, ensuring correct data polarity (negative heading and eye position values for leftward position, positive heading and eye position values for rightward position). Mean and standard deviation time-aligned head angle, horizontal eye position (Fig. 3), and gaze trajectories (Fig. 1*C*) were calculated for Ferrets 1 and 2.

We next quantified the predictivity of horizontal eye position for ferrets' choices at each eye tracker video frame following the onset of the visual stimulus, using the area under the ROC curve. The ROC curve expressed the true positive rate against the false-positive rate of predicting choices across all possible cut points in the distribution of horizontal eye positions (distributions included all trials in all sessions at a given time point following visual stimulus onset per ferret). The area under the ROC curve (AUC) summarized the prediction accuracy across all possible cut points to quantify how well each ferret's horizontal eye position at a given time point following visual stimulus onset predicted their subsequent choice. Curves showing AUC values corresponding to each time frame following visual stimulus onset were generated (Fig. 2*A*). Wilcoxon rank-sum test was used for comparisons between distributions of horizontal eye position at visual stimulus onset before left or right choices (Table 4) and for comparisons of horizontal eye position following left or right choice on the previous trial (Table 4), because eye position distributions were not Gaussian after grouping based on current or previous trial choice.

### Saccade detection

**Figure 4.**
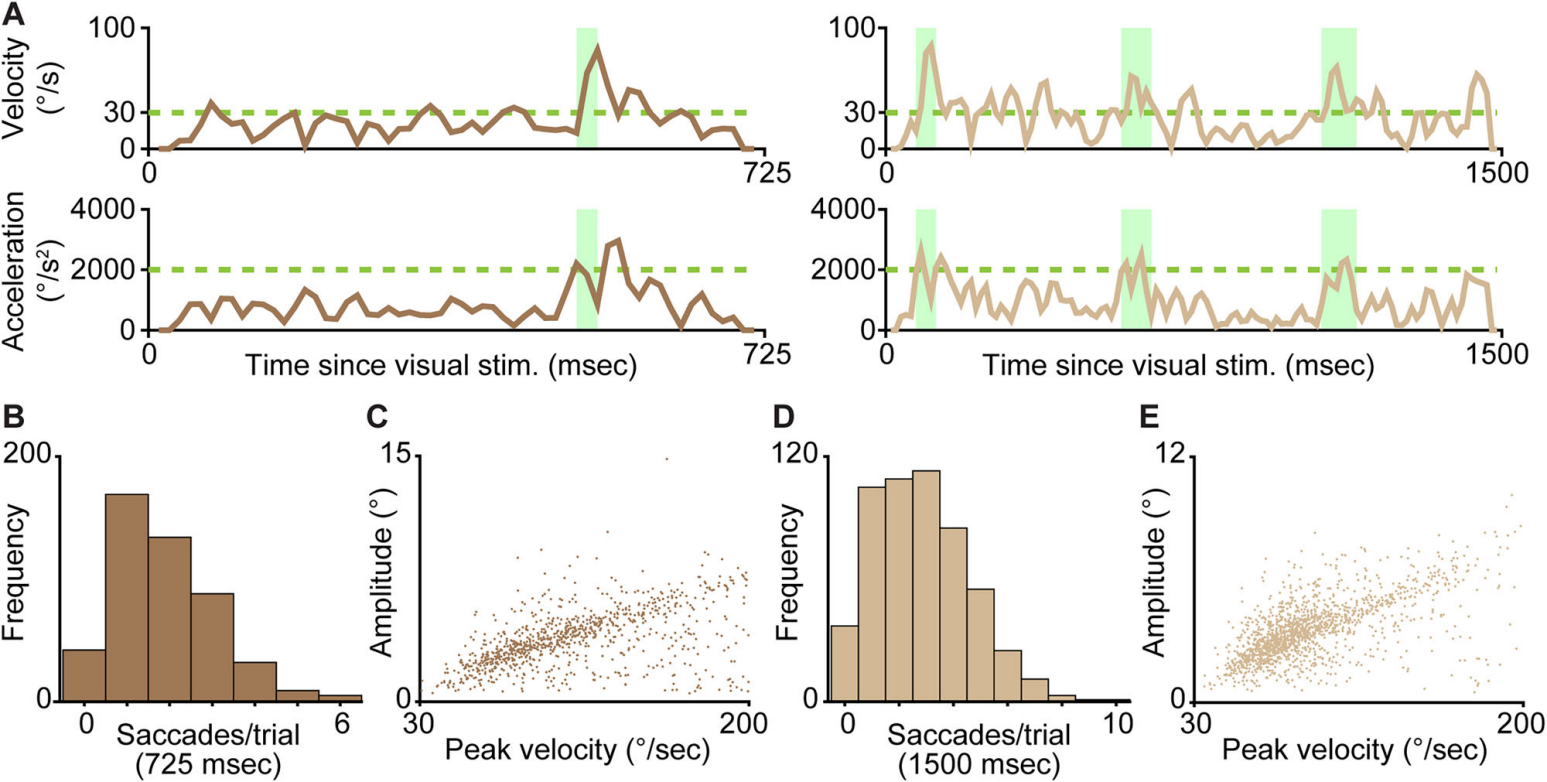
Saccade detection in freely moving ferrets. ***A***, Example saccades detected during visual discriminations from Ferrets 1 and 2. Saccades (green shading) were characterized as pupil movements that surpassed velocity and acceleration thresholds (green dashed lines in top and bottom panels). Note differences in timescale across ferrets. ***B,D***, The number of saccades per visual discrimination trial for Ferret 1 (total trials, 479) and Ferret 2 (total trials, 545). ***C,E***, Saccade amplitude versus peak velocity for Ferrets 1 and 2. Extended Data Figure 4-1 illustrates saccade amplitude, velocity, and pupil size across conditions with associated statistics in Extended Data Figure 4-2.

**Figure 5.**
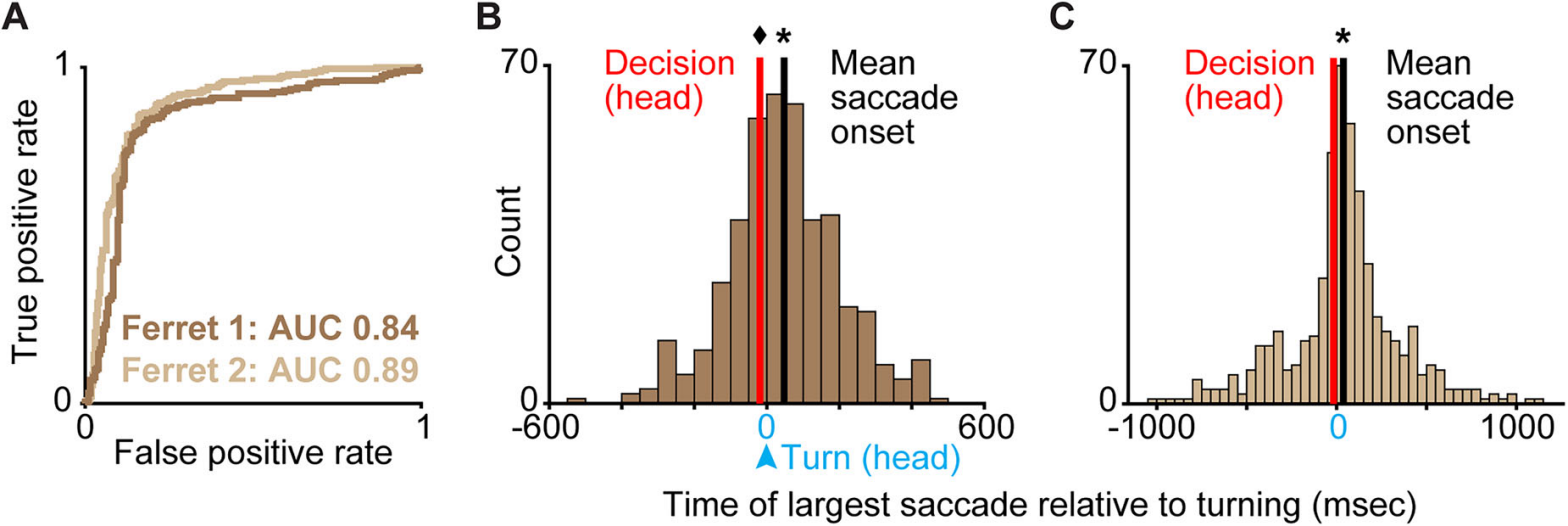
Largest saccades predict ferrets' choices and occur after head turning. ***A***, ROC curves for ferrets' correct choices based on direction of the largest saccade per trial. ***B,C***, Distribution of largest saccade onset time (mean is black line) relative to turning time (time, zero in blue) and shown relative to decision time based on head direction (red line) in Ferrets 1 and 2, respectively. Asterisks indicate significant rightward shift of saccade times relative to turning time; the diamond indicates significant leftward shift of decision time relative to turning time (see Table 2 for statistics and Extended Data Fig. 5-1 for largest saccade distances).

Saccades were measured from horizontal eye position data within the same temporal analysis window described above (unique to each ferret and task). Saccades were detected using available code (Jackson et al., 2008) based on Engbert and Kleigl (2003). Velocity and acceleration of eye movements (akin to first and second derivatives of horizontal eye position over time) occurring within the analysis window were calculated using a five-video-frame window (62.5 ms duration). Periods of time with velocity above 30°/s, peak acceleration above 2,000°/s^2^, and movement amplitude (distance between eye positions at the beginning and end of the period) above 0.5° were marked as saccades (Fig. 4*A*). Saccade onset was defined as the time of the first video frame of the marked saccade period. The mean pupil size across video frames in each saccade period was calculated (Extended Data Fig. 4-1). The quality of saccade detection was evaluated by plotting the peak velocity per saccade against its amplitude (Fig. 4*C*,*E*) because previous studies showed a fixed linear correlation between velocity and amplitude for velocities <200°/s (Engbert and Kleigl, 2003; Jackson et al., 2008). We therefore excluded saccades whose peak velocity was >200°/s, which were rare (0.1–1% of all saccades detected). We also examined the DeepLabCut tracking quality at the onsets and offsets of all saccades and manually excluded rare trials with unreliable eye tracking. We detected saccades (and measured pupil size) when ferrets performed visual discrimination tasks (Fig. 4*B*,*D*), as well as when they freely moved their heads while being handheld (Extended Data Fig. 4-1). Wilcoxon rank-sum tests were used to compare the distributions of saccade number per correct versus incorrect trial for Ferrets 1 and 2 because these distributions were not Gaussian. We quantified the predictivity of horizontal distance/direction of the largest saccade per trial (negative values indicated leftward saccades and positive values indicated rightward saccades) of choice using a similar ROC AUC analysis as described above. The ROC curve expressed the true positive rate against the false-positive rate of predicting choices across all possible cut points in the distribution of saccade distances/directions (distributions included all trials with saccades at a given time point following visual stimulus onset and within the analysis window). The AUC summarized the prediction accuracy across all possible cut points to quantify how well saccade distance/direction predicted ferrets' subsequent choice (Fig. 5*A*). A paired *t* test was used to compare the largest-saccade onset time to the turning time per trial (Fig. 5*B*, Table 2). Unpaired *t* tests were used to compare saccade amplitude, peak velocity, and pupil size between task and handheld conditions (Extended Data Fig. 4-2).

## Results

Our goal was to understand the temporal relationships between two motor actions, head and eye movements, and their predictive power across the continuous process of visual decision-making. To accomplish this goal, we trained five freely moving ferrets to perform visual discrimination tasks using a behavioral box (Fig. 1*A*; modified from Dunn-Weiss et al., 2019). Compared with a head-fixed paradigm, the freely moving paradigm has the advantage of easier training and more natural behavior, at the cost of less control over placement of visual stimuli on the retina (Dunn-Weiss et al., 2019). To mitigate this limitation, we added a corridor with adjacent barriers to the behavioral box (Fig. 1*A*) that forced ferrets to cross the midline at the same position with their heads pointed straight ahead toward the monitor, thus reducing variation in head position and angle across trials. Additionally, we limited the duration of visual stimulus display to the 100–550 ms immediately following midline crossing (Fig. 1*B*) to increase the likelihood that ferrets were facing the center of the monitor during stimulus presentation. Ferrets discriminated the orientation of a single static grating (Ferret 1) or the left or right position of a single static grating on the monitor (Ferret 2) or the relative contrast of two static gratings simultaneously presented at left and right positions on the monitor (Ferrets 2–5). Horizontally displaced gratings were positioned at the eye level (vertical position, 0) and 15° from the center of the monitor. Once trained (see Materials and Methods), ferrets' accuracy at correctly detecting orientation changes, left/right position, or higher contrast gratings across trials varying in difficulty averaged between 72 and 79% (see Table 1 for summary of task performance per ferret). Ferrets completed ∼89–132 trials per session (Table 1) and rarely aborted trials (Ferrets 1–4 aborted 0% of trials and Ferret 5, with the lowest accuracy, aborted 1.9% of trials). The task was designed to avoid aborted trials (see Materials and Methods), and sessions were stopped as soon as ferrets failed to initiate trials for 1 min. Ferrets also differed in the amount of time they took to complete trials, ranging from ∼3 to 9 s on average (Extended Data Fig. 2-1, Table 1). Ferrets 1 and 2 performed trials significantly more slowly while equipped with a head-mounted eye tracker and accompanying vest supporting computing and power for the eye tracker (*p* < 3 × 10^−23^ for both, Wilcoxon rank-sum tests; Extended Data Fig. 2-1*A*; Table 1). On most trials, ferrets followed direct trajectories from the corridor to the chosen reward port, although occasional “wandering” behavior was also observed (Fig. 1*C*, thin lines are example single-trial trajectories). Overall, gaze trajectories did not differ between correct and incorrect trials (Fig. 1*C*, note overlapping thick lines and shaded standard deviation ranges).

10.1523/ENEURO.0124-26.2026.f1-1 Figure 1-1 **Distributions of total trial times for each ferret**. For Ferrets 1 and 2 (top row), data from sessions with and without the eye tracker are color-coded according to the legend. Download Figure 1-1, TIF file.

**Table 1.** Behavior

	Ferret 1	Ferret 1 w/eye tracker	Ferret 2	Ferret 2 w/eye tracker	Ferret 3	Ferret 4	Ferret 5
Accuracy	75 ± 3% (*n* = 74)	73 ± 5% (*n* = 25)	79 ± 6% (*n* = 48)	76 ± 2% (*n* = 15)	74 ± 3% (*n* = 54)	78 ± 5% (*n* = 27)	72 ± 2% (*n* = 32)
Trials performed—all sessions	121 ± 23 (*n* = 74)	132 ± 25 (*n* = 25)	105 ± 12 (*n* = 48)	94 ± 9 (*n* = 15)	101 ± 13 (*n* = 54)	99 ± 9 (*n* = 27)	89 ± 21 (*n* = 32)
Total trial time (seconds)	3.1 ± 2.3	4.1 ± 2.6	4.8 ± 2.5	6.4 ± 4.8	6.2 ± 11	9.1 ± 5.5	3.8 ± 2.3
Trials performed—varying display	100 ± 7 (*n* = 12)	67 ± 21 (*n* = 25)	98 ± 14 (*n* = 7)	108 ± 28 (*n* = 15)	109 ± 12 (*n* = 11)	79 ± 8 (*n* = 22)	56 ± 13 (*n* = 28)
Display time (millisecond)	100–300	400–550	150–400	350–400	200–300	230–280	300–350

Accuracy (mean ± SD) per ferret across all post-training sessions (*n*'s are number of sessions). There were no differences in accuracy with and without the eye tracker for Ferrets 1 or 2 (*p* > 0.09 for both, Wilcoxon rank-sum tests). Number of correct trials completed per session (mean ± SD) across all sessions (*n*'s are number of sessions). Total trial time (mean ± SD) per ferret across all post-training sessions. Ferrets 1 and 2 were significantly slower while wearing the eye tracker (*p* < 3 × 10^−23^ for both, Wilcoxon rank-sum tests). Number of correct trials completed per session (mean ± SD) and range of visual stimulation durations used per ferret during tests of varying grating display duration. *N*'s are total number of post-training testing sessions in which grating display duration varied.

On a subset of sessions per ferret, we varied the duration of the grating display as a secondary test of behavioral performance. When performing these varying display duration trials, ferrets completed 56–109 trials per session on average, using visual stimulus display durations ranging from 100 to 550 ms (Table 1). Ferrets 1 and 2 performed discriminations at 82% accuracy with visual stimulus display duration of ∼325 ms (Fig. 1*D*). These ferrets' performance on varying display duration trials was worse when wearing the eye tracker (Fig. 1*D*, filled squares; Fig. 1*E* illustrates eye tracking). However, accuracy measures for Ferrets 1 and 2 were not significantly different across all sessions when they were wearing the eye tracker versus not (*p* > 0.09 for both, Wilcoxon rank-sum tests). Similarly, their percentages of aborted trials did not change across sessions with and without the eye tracker (Ferret 1, 0% aborted trials in both sets of sessions; Ferret 2, 0% aborted trials without eye tracker and 0.2% aborted trials with eye tracker).

### Head tracking revealed decision time, turning time, and decision biases

To determine whether heading or eye movements predicted behavioral choices in freely moving animals, we first needed to estimate the timing of ferrets' decisions in a manner independent of the type of discrimination task performed or the pace of each ferret (see Materials and Methods). We noted that ferrets' gaze direction tended to orient progressively more toward the reward port of choice over time (Fig. 1*C*). Accordingly, we first tested whether heading direction alone predicted reward port choice. We used an overhead camera to measure ferrets' head movements and applied DeepLabCut (Mathis et al., 2018; Nath et al., 2019; Lauer et al., 2022) to automatically track nose and neck positions per video frame (Fig. 1*A*, inset; Movie 1), from which we calculated head direction relative to the center of the monitor (Fig. 1*A*,*C*). To determine whether head direction predicted choice and to estimate decision timing, we calculated the AUC for time bins corresponding to each video frame after visual stimulus onset for all trials per ferret. Heading was increasingly predictive of choice over time, as AUC values increased over time for all ferrets (Fig. 2*A*; Extended Data Fig. 2-1*A*). Importantly, heading was similarly predictive of choice whether or not ferrets wore eye trackers (Fig. 2*A*). We next defined individual ferrets' decision times as the time to exceed the criterion AUC of 0.875 (see Materials and Methods). Decision times were similar across ferrets (range, 243–325 ms; Table 2) and were also similar to the visual stimulus display duration required to reach 82% accuracy (Fig. 1*D*). The decision time for Ferret 2 while wearing the eye tracker was much slower (600 ms), even though heading predicted choice for Ferret 2 in this condition (Fig. 2*A*, right), consistent with Ferret 2 taking significantly longer to complete trials while wearing the eye tracker (Extended Data Fig. 1-1; Table 1). Importantly, heading was predictive of choice with 95% accuracy within ∼100 ms of the decision time for all ferrets (Fig. 2*A*; Extended Data Fig. 2-1*A*; Table 2), further indicating that the criterion of AUC > 0.875 allowed a good estimate of decision timing across ferrets, independent of task and total trial time or pace.

10.1523/ENEURO.0124-26.2026.f2-1 Figure 2-1 **Head tracking indicates decision time, turning time, and decision bias in Ferrets 3, 4, and 5 (no eye tracker)**. **A**. Area under the ROC curves (AUC) of head direction predictive of correct choice calculated from all trials for 3 additional ferrets. AUC of 0.875 (red dashed line) is the decision criterion from which decision time was assessed (see Methods). **B**. Distributions of head direction at stimulus onset prior to left (L) or right (R) choice per ferret. White lines are medians, the bottom and top edges of the boxes indicate the 25th and 75th percentiles, respectively, whiskers indicate the range of data not considered outliers, and circles are outlier data points. Asterisks indicate significant differences between eventual left/right choices (see Table 3 for statistics). **C**. Distributions of head direction at stimulus onset following previous trial left (L) or right (R) choice per ferret. Conventions as in **B**, see Table 3 for statistics. Download Figure 2-1, TIF file.

**Table 2.** Decision timing

Ferrets	Decision	AUC = 0.95	Turning	Saccade onset
**Ferret 1**	300.0 (*p* = **1.0 × 10^−19^**)	400.0	351.5 ± 130.8 (*n* = 574)	
**Ferret 1 w/eye tracker**	287.5 (*p* = **2.0 × 10^−5^**)	362.5	306.9 ± 101.1 (*n* = 504)	+47.6 ± 154.5 (*p* = **6.0 × 10^−10^**, *n* = 423)
**Ferret 2**	325.0 (*p* = **1.4 × 10^−12^**)	412.5	291.5 ± 112.2 (*n* = 588)	
**Ferret 2 w/eye tracker**	600.0 (*p* = 0.08)	750.0	617.3 ± 233.3 (*n* = 573)	+38.2 ± 316.7 (*p* = **0.007**, *n* = 507)
**Ferret 3**	242.9 (*p* = **0.01**)	285.7	252.2 ± 103.3 (*n* = 783)	
**Ferret 4**	325.0 (*p* = **2.7 × 10^−35^**)	412.5	369.9 ± 128.1 (*n* = 1,327)	
**Ferret 5**	262.5 (*p* = **4.0 × 10^−24^**)	337.5	301.8 ± 111.2 (*n* = 756)	

Time (all in millisecond) of decision (AUC, 0.875); of AUC, 0.95; of turning following stimulus onset (mean ± SD); and of the largest-saccade onset relative to turning time (mean ± SD), where positive values indicate saccade onset after turning time. *N*'s are total number of trials. *p* values of decision time (one-sample *t* test) were calculated for comparisons of turning time per trial to decision time. *p* values of saccade onset time (paired *t* test) were calculated for comparisons of the largest-saccade onset time and turning time per trial. Bold *p* values are significant.

Having confirmed that heading direction was predictive of ferrets' behavioral choices, we next asked whether we could detect choice-indicative head turns per trial. Using the same heading direction data, we fit bilinear curves to head direction over time per trial and defined per-trial turning time from the point of inflection in each curve (Fig. 2*B*). Mean turning times were also similar across ferrets (range, 252–370 ms except Ferret 2 with eye tracker at 617 ms) and turning times followed decision times per ferret except for Ferret 2 without eye tracker (Table 2). While behavioral variability could have been due to changes in pace (Extended Data Fig. 1-1) and/or wandering behavior, e.g., premature turning followed by reorienting (Fig. 1*C*, thin lines), changes in the ordering of decision time and turning time across conditions could also be explained by alterations of decision biases across conditions.

We therefore explored biases in ferrets' decision-making by examining heading at the beginning of the decision-making process. We first asked whether ferrets' head direction at the onset of the visual stimulus display biased their eventual left or right choices. Across all ferrets (and in conditions without and while wearing the eye tracker), initial heading direction at stimulus onset was significantly different preceding eventual left versus right choices (Fig. 2*C*; Extended Data Fig. 2-1*B*), suggesting biasing of choices by initial heading. These biases were small and usually predicted choice consistent with ferrets' initial heading (Tables 3, 4). We next asked whether ferrets' previous choices, whether correct or incorrect, biased their heading on the next trial, i.e., whether they adopted “stay” or “switch” strategies. Again, all ferrets' initial heading at stimulus onset significantly differed when following prior left versus right choices (Fig. 2*D*; Extended Data Fig. 2-1*C*). These biases in Ferrets 1 and 2 were not consistent with and without the eye tracker. Ferrets generally showed switch biases in their heading following prior choices (Tables 3, 4). Ferrets may have adopted switching strategies because the trial conditions were pseudorandomized to produce an approximately equal number of left- and right-choice trials. In other words, consecutive trials were slightly more likely to have opposite than same side correct outcomes. Importantly, although heading biases were significant, they were small, on the order of 2° (Tables 3, 4), and did not severely limit ferrets' daily performance (Table 1). Furthermore, differences in heading for left versus right choices at the decision time and at turning times were much larger, on the order of 10–20° across all ferrets, compared with small (∼2°) biases in heading at stimulus onset (Tables 3, 4).

**Table 3.** Heading

Ferrets	Side of choice	Onset	*p* value	Decision	Turning
**Ferret 1**	Left	0.4 ± 4.7 (*n* = 154)	**0.047**	−9.7 ± 8.4	−12.0 ± 14.0
	Right	−0.5 ± 3.8 (*n* = 423)		4.7 ± 9.0	3.0 ± 12.2
	Post L	−0.5 ± 3.8 (*n* = 288)	0.09		
	Post R	0.0 ± 4.3 (*n* = 289)			
**Ferret 2**	Left	−2.0 ± 4.7 (*n* = 299)	**5.0 × 10^−6^**	−3.6 ± 9.2	2.4 ± 14.3
	Right	−0.2 ± 3.7 (*n* = 305)		11.6 ± 9.3	3.9 ± 9.6
	Post L	−0.8 ± 4.6 (*n* = 300)	**0.02**		
	Post R	−1.3 ± 4.1 (*n* = 304)			
**Ferret 3**	Left	−6.0 ± 3.9 (*n* = 371)	**8.1 × 10^−4^**	−11.9 ± 6.6	−6.0 ± 8.2
	Right	−5.0 ± 3.9 (*n* = 439)		5.1 ± 11.9	5.0 ± 15.0
	Post L	−4.7 ± 4.0 (*n* = 390)	**6.4 × 10^−8^**		
	Post R	−6.2 ± 3.8 (*n* = 420)			
**Ferret 4**	Left	−4.8 ± 6.6 (*n* = 685)	**7.6 × 10^−11^**	−13.1 ± 6.3	−14.3 ± 8.3
	Right	−2.1 ± 7.6 (*n* = 646)		−0.3 ± 9.0	−4.0 ± 10.3
	Post L	−2.9 ± 7.6 (*n* = 678)	**0.001**		
	Post R	−4.2 ± 6.7 (*n* = 653)			
**Ferret 5**	Left	−3.2 ± 6.0 (*n* = 325)	**1.4 × 10^−8^**	−16.5 ± 7.6	−18.0 ± 12.0
	Right	−0.7 ± 6.2 (*n* = 452)		0.7 ± 11.6	−1.9 ± 13.2
	Post L	−0.5 ± 6.0 (*n* = 345)	**1.7 × 10^−7^**		
	Post R	−2.7 ± 6.3 (*n* = 432)			

Head direction (mean ± SD) in degrees; negative indicates leftward, and positive indicates rightward angles, at visual stimulus onset, decision time, and turning time, separated by subsequent left or right choice or by previous left or right choice (Post L/Post R). *N*'s are total number of trials. *p* values (Wilcoxon rank-sum tests) were calculated for comparisons of head directions and eye positions at stimulus onset for left versus right choices. Bold *p* values are significant.

**Table 4.** Heading with eye tracking

Ferrets	Side of choice	Onset				Decision		Turning	
		Head	*p*	Eye	*p*	Head	Eye	Head	Eye
Ferret 1 w/eye tracker	Left	4.3 ± 3.0 (*n* = 235)	**3.7 × 10^−8^**	−0.5 ± 2.1 (*n* = 213)	**0**.**005**	−3.0 ± 7.3	−2.4 ± 2.4	1.4 ± 11.3	−1.0 ± 2.0
	Right	5.2 ± 3.5 (*n* = 287)		−0.1 ± 2.0 (*n* = 266)		7.7 ± 6.4	−0.2 ± 2.4	6.2 ± 9.0	−0.1 ± 2.0
	Post L	5.6 ± 3.1 (*n* = 241)	**4.0 × 10^−8^**	0.2 ± 1.8 (*n* = 227)	**1.4 × 10^−7^**				
	Post R	4.1 ± 3.3 (*n* = 281)		−0.8 ± 2.2 (*n* = 252)					
Ferret 2 w/eye tracker	Left	−2.5 ± 3.7 (*n* = 304)	**1.9 × 10^−4^**	0.1 ± 1.6 (*n* = 285)	1.0	−10.0 ± 13.4	−1.1 ± 1.8	−3.7 ± 17.6	−0.6 ± 1.6
	Right	−1.2 ± 3.7 (*n* = 270)		0.0 ± 1.5 (*n* = 260)		11.9 ± 12.7	0.9 ± 1.9	4.4 ± 14.4	0.2 ± 1.3
	Post L	−1.8 ± 3.8 (*n* = 276)	0.9	0.1 ± 1.6 (*n* = 260)	0.3				
	Post R	−1.9 ± 3.8 (*n* = 298)		0.0 ± 1.6 (*n* = 285)					

Head direction (mean ± SD) in degrees; negative indicates leftward, and positive indicates rightward, and eye horizontal direction (mean ± SD) in degrees, normalized to mean eye position before stimulus onset; negative indicates leftward, and positive indicates rightward, each at stimulus onset, decision time, and turning time, and separated by subsequent left or right choice or by previous left or right choice (Post L/Post R). *N*'s are total number of trials. *p* values (unpaired *t* tests) were calculated for comparisons of head directions and eye positions at stimulus onset for left versus right choices. Bold *p* values are significant.

### Heading predicted choice more reliably than eye position

Having demonstrated that heading was predictive of ferrets' behavioral choices as well as their biases and task strategies, we next asked whether eye movements were similarly predictive of choices. Ferrets 1 and 2 were fitted with head-mounted eye trackers and accompanying vests to support onboard electronics and batteries. Wearing this apparatus significantly slowed these ferrets down (Extended Data Fig. 1-1; Table 1) and altered their performance on a subset of sessions with varying stimulus display duration trials (Fig. 1*D*). However, overall accuracy and number of completed trials across all sessions did not differ when Ferrets 1 and 2 wore the eye tracker versus not (Table 1). Time-aligned head angle and horizontal eye position trajectories for Ferrets 1 and 2 (all from sessions with eye trackers) suggested that heading and eye position were aligned over the course of decision-making (Fig. 3*A*,*B*). We observed that ferrets often made horizontal eye movements, including saccades, in the same direction as their eventual choice ports (Fig. 3*A*,*B*). Furthermore, choice-directed horizontal eye movements appeared to occur around the time that heading angled toward chosen ports (Fig. 3*A*,*B*, rightmost). We therefore sought to quantify whether eye movements on their own were choice-orientated and/or similarly predictive of choice as heading. Eye tracking camera video frames were analyzed using DeepLabCut to automatically label and track the pupil and eyelid outline (Fig. 1*E*; Movie 2) from which their relative positions were computed per frame to estimate the horizontal and vertical position of the pupil. We first asked whether raw horizontal eye position was predictive of behavioral choices using the same AUC analysis described above. In both ferrets, horizontal eye position was maximally predictive of choice after the decision time (at heading AUC > 0.875) and was never as strongly predictive of choice compared with heading (Fig. 2*A*, compare black curves for horizontal eye position to brown curves for heading). We next explored horizontal eye position around turning time per trial, to assess whether left or right eye movements preceded or followed left or right head turns, i.e., to test whether horizontal eye movements were choice-oriented or otherwise. Horizontal eye position was directed toward ferrets’ subsequent choices: eyes moved left for leftward choices and vice versa (Extended Data Fig. 3-1; Table 4), consistent with heading (Fig. 3*A*,*B*). In both ferrets, horizontal eye movements peaked after head turning time (Extended Data Fig. 3-1). Small, compensatory eye movements in the opposite direction often followed choice-directed eye movements (Extended Data Fig. 3-1), but choice-oriented eye movements were much larger and always occurred earlier. Notably, horizontal eye position only reflected prior trial choice or subsequent choice bias at stimulus onset for Ferret 1, and again these biases were small (<0.5°), smaller than eye position differences at decision and turning times (which were on the order of 1–2°; Table 4).

10.1523/ENEURO.0124-26.2026.f3-1 Figure 3-1 **Eye position relative to turning time.** Mean eye position (degrees in horizontal direction, relative to the center of the eye) aligned to the turning time (blue line) per trial in Ferret 1 (**A**) and Ferret 2 (**B**). Green and orange curves show data prior to right and left choice, respectively. Shaded areas show SEMs. Note differences in timescales across ferrets. Download Figure 3-1, TIF file.

### Saccade detection in freely moving ferrets

The large, choice-oriented horizontal eye movements ferrets made around the time of their decisions (Fig. 3*A*,*B*, leftmost) suggested ferrets were making saccades. We therefore detected saccades based on synchronous changes in velocity and acceleration of eye position traces (Fig. 4*A*; see Materials and Methods). At least one saccade was detected during most visual discrimination trials for both Ferrets 1 and 2 (Fig. 4*B*,*D*). Both ferrets made similar numbers of saccades on correct and incorrect trials [Ferret 1, mean number of saccades on correct trials, 1.8 ± 1.3; on incorrect trials, 1.6 ± 1.2 (725 ms window); Ferret 2, mean number of saccades on correct trials, 2.8 ± 1.9; on incorrect trials, 2.9 ± 1.9 (1,500 ms window)]. Saccades measured in both ferrets demonstrated a linear relationship between peak velocity and amplitude (Fig. 4*C*,*E*), consistent with saccades measured in humans (Engbert and Kleigl, 2003; Jackson et al., 2008). Both ferrets made larger-amplitude and thus higher-velocity saccades while handheld compared with saccades measured during visual discrimination trials in the behavioral box (Extended Data Figs. 4-1, 4-2). The pupil size was also significantly larger while both ferrets were performing visual discrimination trials compared with being handheld (Extended Data Figs. 4-1*A*,*D*,*E*,*H*, 4-2). Thus, the eye tracker was not limited to detecting small saccades. Larger pupil diameters during trials also suggest ferrets were engaged with the tasks.

10.1523/ENEURO.0124-26.2026.f4-1 Figure 4-1 **Eye tracking in Ferrets 1 and 2 while hand-held**. **A**. Example video frame of eye tracking in Ferret 1. The boundaries of the pupil and the eyelid were labeled automatically using DeepLabCut and fit with ellipses (red). **B**-**D**. Saccade amplitude (**B**), peak velocity (**C**), and pupil size (**D**) in Ferret 1 when performing visual discrimination tasks compared to being held. Black lines are medians, the bottom and top edges of the boxes indicate the 25th and 75th percentiles, respectively, whiskers indicate the range of data not considered outliers, and circles are outlier data points. Asterisks indicate significant differences across conditions (see Figure 4-2 for statistics). **E**-**H**. Pupil tracking and saccade and pupil size data for Ferret 2, conventions as in **A**-**D**. Download Figure 4-1, TIF file.

10.1523/ENEURO.0124-26.2026.f4-2 Figure 4-2 **Saccades.** Saccade amplitude (degrees in any direction), peak velocity (deg/sec in any direction), and pupil size (mean ± SD) were measured while ferrets performed a task or were hand-held. N’s are total number of trials. P values (unpaired t-tests) were calculated for comparisons of each metric across the two conditions. Bold P values indicate significant differences. Download Figure 4-2, DOCX file.

### Largest saccades predicted choices and occurred after head turning

Because ferrets made horizontal eye movements, including saccades, in the same direction as their eventual choice ports and around the same time as heading angled toward choice ports (Fig. 3), we next tested whether horizontal saccades predicted ferrets' choices and computed saccade timing relative to head turning. We again employed an ROC analysis (see Materials and Methods) to test whether the largest horizontal saccades per trial were predictive of ferrets' choices. For Ferrets 1 and 2, the largest horizontal saccades per trial predicted correct left or right choices at AUC values of 0.84 and 0.89 (Fig. 5*A*), comparable to the predictability of choice from heading at the decision time. The largest saccades were in the same direction as animals' choices (Extended Data Fig. 5-1), consistent with horizontal eye position measurements (Fig. 3, Extended Data Fig. 3-1), suggesting these saccades were not vestibulo-ocular reflexes caused by head turning. Subsequent smaller saccades in the opposite direction of the initial saccade and head turn were also observed (Extended Data Fig. 3-1*A*), suggesting ferrets also make counter-rotational, compensatory saccades to stabilize gaze, consistent with prior observations in carnivores and primates (Blakemore and Donaghy, 1980; Guitton et al., 1990; Galiana and Guitton, 1992; Singh et al., 2025; Wallace et al., 2025). Finally, we asked when the largest saccades per trial occurred relative to the head turning time per trial. For both ferrets, the largest horizontal saccades happened significantly later than the head turning time (Fig. 5*B*,*C*). For Ferret 1, the distribution of largest horizontal saccade times was also shifted to be significantly later than the decision time (Fig. 4*B*). Thus, although saccades, as well as horizontal eye position, were predictive of behavioral choice, they were less predictive than heading. Additionally, saccades tended to occur after heading indicated behavioral choice.

10.1523/ENEURO.0124-26.2026.f5-1 Figure 5-1 **Largest saccades**. Horizontal distance (mean ± SD) in degrees, negative indicates leftward and positive indicates rightward, of the largest amplitude saccades in left- or right-choice trials. N’s are total number of trials. Download Figure 5-1, DOCX file.

## Discussion

Decision-making is a continuous process that develops over time. Tracking eye and head movements in freely moving subjects may provide noninvasive readouts of a more natural decision process. In this study, we simultaneously tracked head and eye movements in freely moving ferrets performing visual discrimination tasks. Ferrets usually took three or more seconds to complete a trial in our behavioral setup (Extended Data Fig. 2-1, Table 1). This relatively long trial duration was beneficial in studying how decision-making develops over time. We found that heading reliably indicated ferrets’ decision formation and also revealed their decision biases and choice strategies at visual stimulus onset. Ferrets' decision times, estimated statistically based on heading angle, occurred ∼300 ms after visual stimulus onset and were followed shortly thereafter by choice-oriented horizontal eye movements, including saccades. Like heading, horizontal saccades also predicted ferrets' behavioral choices, although saccades occurred after heading redirections associated with decisions. These results demonstrate that freely moving, highly visual mammals orient their heads and then their eyes when selecting a behaviorally relevant target during visual discrimination tasks.

Ferrets performed two-alternative forced choice tasks, so the number of different observable behavioral patterns was limited. It was expected that ferrets would eventually orient their heads and eyes toward chosen reward ports. What was unexpected, and not necessarily predicted, was the timing of orienting heading and eye movements. First, ferrets were decisive. They headed to their chosen port as soon as they exited the corridor, shortly after stimulus onset, rather than continuing straight toward the monitor for a longer look before deciding. They seldom demonstrated wandering behaviors, even on incorrect trials, and a small, but significant, portion of their decisiveness was due to biases present at the first visual stimulus presentation frame. Notably, all five ferrets made decisions quickly, even though their total trial durations varied substantially (Table 1, Extended Data Fig. 2-1). Such decisive behavior in a freely moving animal was unexpected, especially due to known variability within and across individual subjects in terms of motivation and task engagement. Second, ferrets' saccades usually occurred after they had viewed stimuli (which were often laterally displaced on the monitor) and reached a decision. Rather than utilizing saccades to gather visual information to inform their decision, they captured sufficient information in a short time window (∼300 ms), made their decision, and then utilized saccades to orient toward a reward port. This strategy of utilizing central and peripheral visual inputs to make quick decisions at the expense of acuity was also unexpected and not necessarily predicted for a highly visual mammal. We assumed that scanning visual targets would be optimal for target selection, but our results suggest animals instead utilized a cost–benefit strategy that minimized visual searching and the time to reward at the expense of accuracy when they were free to determine the pace and method of decision-making.

The reward ports in our behavioral box were ∼30° away from the center of the monitor when viewed from the midline. This is a larger distance relative to saccade distances utilized in most visual decision-making tasks in which humans or NHPs typically saccade from an initial fixation spot to an answer target ∼5–10° away on a monitor (Gold and Shadlen, 2007). We did not detect 30° saccades in ferrets in the task or while handheld (Extended Data Fig. 4-1). Instead, ferrets reoriented toward chosen reward ports using a combination of head turns and choice-oriented eye movements (Figs. 1*C*, 3), consistent with prior work showing that larger gaze shifts involve combined head and eye movements (Blakemore and Donaghy, 1980; Zangemeister and Stark, 1982; Guitton et al., 1984; Freedman and Sparks, 1997). Ferrets' decision-predictive eye movements were not optokinetic reflexive movements, as they were in the same direction as choices and head turns (Table 4; Extended Data Figs. 3-1, 5-1). We did detect counter-rotational eye movements following decision-predictive saccades (Extended Data Fig. 3-1), consistent with prior observations of gaze stabilization eye movements in non-head-restrained animals (Blakemore and Donaghy, 1980; Guitton et al., 1990; Galiana and Guitton, 1992; Singh et al., 2025). The combination of head turning and saccades used by ferrets to orient toward the reward ports is consistent with a “saccade-and-fixate” pattern where saccades are made in the same direction as head movements to shift gaze onto the new visual target, a behavioral pattern conserved across the animal kingdom (Galiana and Guitton, 1992; Freedman, 2008; Land, 2019; Meyer et al., 2020; Michaiel et al., 2020; Skyberg and Niell, 2024; Singh et al., 2025; Wallace et al., 2025). Here, while saccade direction was predictive of choice, saccades followed heading reorienting, supporting the notion that visual mammals first turn their heads and then their eyes to orient toward relevant targets more than a few degrees from their initial gaze position (Blakemore and Donaghy, 1980; Zangemeister and Stark, 1982; Guitton et al., 1984; Freedman and Sparks, 1997). In humans, head movements contribute more toward gaze shifts when targets are predictable, often preceding saccades in these conditions (Bizzi et al., 1972; Moschner and Zangemeister, 1993).

It is important to note that eye tracking in freely moving animals is limited in several ways. First, precise calibration of an eye tracker requires the animal to perform a calibration routine involving fixation on an array of displayed visual stimuli such that pupil position corresponding to each fixation is calculated in real time. Our ferrets were not head restrained or fixation trained, and therefore we could not perform such a routine. Some groups have utilized indirect methods to “calibrate” eye and head tracking cameras relative to one another, i.e., while cameras are mounted on a fixed surface and not attached to animals (Wallace et al., 2013, Supplemental Information) or by altering camera position relative to the eye of a head-fixed animal (Zoccolan et al., 2010). These sophisticated approaches still approximate true eye-in-head position and/or were not applicable here due to lack of head restraint. Accordingly, because calibration was not possible in our setup, we did not attempt to analyze information about stimuli at the center of ferrets' gaze. We assumed, based on all five ferrets' performance in the tasks, that they were viewing visual stimuli in order to make their decisions, and we relied on recent findings that ferrets tend to maintain visual targets at or near the area centralis, independent of body movement trajectories (Wallace et al., 2025). Instead, we analyzed measurements not subject to calibration confounds, namely, horizontal direction of eye movements and timing of saccades.

A second limitation is monocular eye tracking. In this study, a single camera could not reliably detect pupils in both eyes, and our setup could not accommodate two head-mounted eye trackers. Wallace et al. (2025) tracked eye position simultaneously in both eyes in freely moving ferrets, and while they did not quantify conjugation of binocular eye movements, eye movements appeared correlated in ferrets. Binocular eye movements are strongly correlated in head-stabilized humans, but binocular eye movements are only weakly correlated in freely moving mice (Meyer et al., 2020). Accurate pupil tracking in ferrets requires adjustments of the positions and angles of the IR-reflective mirror and camera so that the camera captures the frontal view of the eye. We attempted to track both eyes with a single camera but could not achieve reliable pupil tracking in both eyes. Furthermore, using two cameras requires an additional Raspberry pi computer and battery, which could further alter performance beyond what we observed with our single-camera system. Dual eye tracking may be achieved with a tethering system and/or further miniaturization of the camera/mirror/headpost as well as the on-vest electronics. Indeed, Wallace et al. (2025) implemented technological advances to achieve binocular eye tracking in freely moving ferrets. So, we may continue to learn more about correlated binocular eye movements in freely moving ferrets and other species.

It is important to consider constraints various devices place on animals and the possibility that their free movement and behavioral performance may become impacted. We observed adequate overall performance across ferrets: ∼100 correctly completed trials per session, consistent with prior reports of ferrets performing similar tasks (Dunn-Weiss et al., 2019). However, wearing the eye tracker and associated accessories significantly slowed ferrets down (Extended Data Fig. 2-1) and reduced ferrets’ performance on a subset of trials in which the visual stimulus display duration varied (Fig. 1*D*). Wearing the eye tracker did not change the total number of trials performed or accuracy across all sessions, and heading was still predictive of choices even when ferrets moved more slowly. Nonetheless, considering that some animals may show performance changes or slowing of movement when wearing equipment, is eye tracking necessary for freely moving behavior studies? Tracking heading provided an earlier and more reliable predictor of ferrets' decisions compared with saccades and saccades did not occur on all trials. If optimal task performance is required, it may be best to forgo eye tracking, especially when smaller animals (e.g., female ferrets) are used. However, eye tracking provides additional information, like pupil size, associated with alertness (Stitt et al., 2018).

An additional unexpected outcome of this study was the ability to detect ferrets' decision biases using heading measurements. All ferrets had decision biases at visual stimulus onset, revealed by heading (Fig. 2). Ferrets likely adopted switch biases, expectations that the next reward would be at the previously unselected port, to avoid hesitation and reduce the time until the next reward, a speed-accuracy trade-off approach. Ferrets were as decisive when choosing correctly and when making the wrong decision: gaze trajectories were similarly direct on correct and incorrect trials. Ferrets were engaged in nearly continuous locomotion while not at ports, and their total trial times, though varied across animals, suggested minimal time wasting. Ferrets also showed small but significant biases based on their initial heading at visual stimulus onset (Fig. 2*D*; Extended Data Fig. 2-1*C*). Thus, ferrets utilized a combination of prior knowledge and expectation along with current visual information to form their decisions and act quickly.

In addition to revealing biases and strategies, the freely moving paradigm enabled observation of behavioral variability within and across animals. Ferret 2 performed the same task as Ferrets 3–5 with similar accuracy and numbers of completed trials per session. Like Ferret 1, Ferret 2 moved significantly more slowly while wearing the eye tracker. Even so, Ferret 2 took much longer to make decisions while wearing the eye tracker (Table 2), such that its head turning was less predictive of eventual choice during these sessions. Curiously, Ferret 2's turning time preceded its decision time on sessions when it was not wearing the eye tracker, while none of the other ferrets demonstrated this pattern. Without the eye tracker, Ferret 2 may have been prone to rushing (trading speed for accuracy, with switch bias). When the eye tracker slowed Ferret 2 down, its switch bias went away (compare Tables 3, 4), and its decision time preceded its turning time, as for the other ferrets. This suggests strategies for decision-making are flexible, depending on conditions and/or context. Examining decision-making in freely moving animals provides a unique paradigm to study this process and its variability. Further technological improvements, like further miniaturization of devices, chronically implanted electrodes, and wireless signal transmission, combined with more naturalistic freely moving setups will shed more light onto the neuronal mechanism underlying decision-making dynamics and variability during behavior.

In summary, we examined freely moving ferrets performing visual discrimination tasks while tracking their head and eye movements. Heading was predictive of choices as well as biases and decision strategies. While choice-oriented saccades also predicted choices, they were less predictive than heading and occurred after head turning. Our findings show that when unrestrained, highly visual mammals orient first with their heads and then with their eyes when behaviorally relevant targets are more than a few degrees from initial gaze. Furthermore, we established a unique paradigm to probe the continuous process of visual decision-making in a more naturalistic manner. Our findings also suggest there are multiple, flexible steps of decision-making for ferrets performing visual discriminations, corroborating the notion that decision-making is a continuous and evolving process (Wispinski et al., 2020; Spering, 2022).

## Data Availability

Data that support the findings will be posted to Neuroscience Data Interface following standardization and is currently available upon request. Custom code is also available here: https://github.com/BriggsNeuro.

## Synthesis

Reviewing Editor: David Schoppik, New York University Grossman School of Medicine Decisions are customarily a result of the Reviewing Editor and the peer reviewers coming together and discussing their recommendations until a consensus is reached. When revisions are invited, a fact-based synthesis statement explaining their decision and outlining what is needed to prepare a revision will be listed below. The following reviewer(s) agreed to reveal their identity: NONE. Note: If this manuscript was transferred from JNeurosci and a decision was made to accept the manuscript without peer review, a synthesis may not be available.

The revised manuscript addresses all of the reviewers' concerns, to the extent possible, given methodological limits. The text now adequately conveys these limits and, as such, is considerably stronger.

## References

[B1] Baker M (2013) Neuroscience: through the eyes of a mouse. Nature 502:156–158. 10.1038/502156a 24108031

[B2] Bizzi E, Kalil RE, Morasso P (1972) Two modes of active eye-head coordination in monkeys. Brain Res 40:45–48. 10.1016/0006-8993(72)90104-7 4624490

[B3] Blakemore C, Donaghy M (1980) Co-ordination of head and eyes in the gaze changing behavior of cats. J Physiol 300:317–335. 10.1113/jphysiol.1980.sp013164 7381790 PMC1279357

[B4] Brainard DH (1997) The psychophysics toolbox. Spat Vis 10:433–436. 10.1163/156856897X00357 9176952

[B5] Drager UC (1974) Autoradiography of tritiated proline and fucose transported transneuronally from the eye to the visual cortex in pigmented and albino mice. Brain Res 82:284–292. 10.1016/0006-8993(74)90607-6 4441894

[B6] Dunn-Weiss E, Nummela SU, Lempel AA, Law JM, Ledley J, Salvino P, Nielsen KJ (2019) Visual motion and form integration in the behaving ferret. eNeuro 6:1–19. 10.1523/ENEURO.0228-19.2019 PMC6709227 31371456

[B7] Eastman KM, Huk AC (2012) PLDAPS: a hardware architecture and software toolbox for neurophysiology requiring complex visual stimuli and online behavioral control. Front Neuroinform 6:1–10. 10.3389/fninf.2012.00001 22319490 PMC3269100

[B8] Engbert R, Kleigl R (2003) Microsaccades uncover the orientation of covert attention. Vision Res 43:1035–1045. 10.1016/S0042-6989(03)00084-1 12676246

[B9] Freedman EG (2008) Coordination of the eyes and head during visual orienting. Exp Brain Res 190:369–387. 10.1007/s00221-008-1504-8 18704387 PMC2605952

[B10] Freedman EG, Sparks DL (1997) Eye-head coordination during head-unrestrained gaze shifts in rhesus monkeys. J Neurophysiol 77:2328–2348. 10.1152/jn.1997.77.5.2328 9163361

[B11] Galiana HL, Guitton D (1992) Central organization and modeling of eye-head coordination during orienting gaze shifts. Ann N Y Acad Sci 656:452–471. 10.1111/j.1749-6632.1992.tb25228.x 1599162

[B12] Gallivan JP, Chapman CS, Wolpert DM, Flanagan JR (2018) Decision-making in sensorimotor control. Nat Rev Neurosci 19:519–534. 10.1038/s41583-018-0045-9 30089888 PMC6107066

[B13] Gold JI, Shadlen MN (2007) The neural basis of decision making. Ann Rev Neurosci 30:535–574. 10.1146/annurev.neuro.29.051605.113038 17600525

[B14] Guitton D, Douglas RM, Volle M (1984) Eye-head coordination in cats. J Neurophysiol 52:1030–1050. 10.1152/jn.1984.52.6.1030 6335170

[B15] Guitton D, Munoz DP, Galiana HL (1990) Gaze control in the cat: studies and modeling of the coupling between orienting eye and head movements in different behavioral tasks. J Neurophysiol 64:509–531. 10.1152/jn.1990.64.2.509 2213129

[B16] Hollensteiner KJ, Pieper F, Engler G, Konig P, Engel AK (2015) Crossmodal integration improves sensory detection thresholds in the ferret. PLoS One 10:1–10. 10.1371/journal.pone.0124952 PMC4430165 25970327

[B17] Hupfeld D, Distler C, Hoffmann KP (2006) Motion perception deficits in albino ferrets (*Mustela putorius furo*). Vision Res 46:2941–2948. 10.1016/j.visres.2006.02.020 16647737

[B18] Hupfeld D, Distler C, Hoffmann KP (2007) Deficits in visual motion perception and optokinetic nystagmus after posterior suprasylvian lesions in the ferret (*Mustela putorius furo*). Exp Brain Res 182:509–523. 10.1007/s00221-007-1009-x 17593360

[B19] Jackson CA, Hickey TL (1985) Use of ferrets in studies of the visual system. Lab Anim Sci 35:211–215. 3894786

[B20] Jackson S, Cummins F, Brady N (2008) Rapid perceptual switching of a reversible biological figure. PLoS One 3:1–10. 10.1371/journal.pone.0003982 PMC2601034 19093003

[B21] Kimmel DL, Mammo D, Newsome WT (2012) Tracking the eye non-invasively: simultaneous comparison of the scleral search coil and optical tracking techniques in the macaque monkey. Front Behav Neurosci 6:1–17. 10.3389/fnbeh.2012.00049 22912608 PMC3418577

[B22] Kleiner M, Brainard DH, Pelli D (2007) What’s new in Psychtoolbox-3? Perception 36:1–89.

[B23] Land MF (2019) The evoluation of gaze shifting eye movements. In: *Current topics in behavioral neurosciences* (Hodgson T, ed), pp 3–11. Cham, Switzerland: Springer Nature. 10.1007/7854_2018_60 30120752

[B24] Lauer J, et al. (2022) Multi-animal pose estimation, identification and tracking with DeepLabCut. Nat Methods 19:496–504. 10.1038/s41592-022-01443-0 35414125 PMC9007739

[B25] Law MI, Zahs KR, Stryker MP (1988) Organization of primary visual cortex (area 17) in the ferret. J Comp Neurol 278:157–180. 10.1002/cne.902780202 3068264

[B26] Leinonen H, Tanila H (2018) Vision in laboratory rodents - tools to measure it and implications for behavioral research. Behav Brain Res 352:172–182. 10.1016/j.bbr.2017.07.040 28760697

[B27] Mandrekar JN (2010) Receiver operating characteristic curve in diagnostic test assessment. J Thorac Oncol 5:1315–1316. 10.1097/JTO.0b013e3181ec173d 20736804

[B28] Mao D, Avila E, Caziot B, Laurens J, Dickman JD, Angelaki DE (2021) Spatial modulation of hippocampal activity in freely moving macaques. Neuron 109:3521–3534. 10.1016/j.neuron.2021.09.032 34644546 PMC8571030

[B29] Mathis A, Mamidanna P, Cury KM, Abe T, Murthy VN, Mathis MW, Bethge M (2018) DeepLabCut: markerless pose estimation of user-defined body parts with deep learning. Nat Neurosci 21:1281–1289. 10.1038/s41593-018-0209-y 30127430

[B30] Meyer AF, Poort J, O’Keefe J, Sahani M, Linden J (2018) A head-mounted camera system integrates detailed behavioral monitoring with multichannel electrophysiology in freely moving mice. Neuron 100:46–60. 10.1016/j.neuron.2018.09.020 30308171 PMC6195680

[B31] Meyer AF, O’Keefe J, Poort J (2020) Two distinct types of eye-head coupling in freely moving mice. Curr Biol 30:2116–2130. 10.1016/j.cub.2020.04.042 32413309 PMC7284311

[B32] Michaiel AM, Abe ETT, Niell C (2020) Dynamics of gaze control during prey capture in freely moving mice. Elife 9:1–13. 10.7554/eLife.57458 PMC7438109 32706335

[B33] Milton R, Shahidi N, Dragoi V (2020) Dynamic states of population activity in prefrontal cortical networks of freely-moving macaque. Nat Commun 11:1–10. 10.1038/s41467-020-15803-x 32327660 PMC7181779

[B34] Moschner C, Zangemeister WH (1993) Preview control of gaze saccades: efficacy of prediction modulates eye-head interaction during human gaze saccades. Neurol Res 15:417–432. 10.1080/01616412.1993.11740176 7907412

[B35] Nath T, Mathis A, Chen AC, Patel AA, Bethge M, Mathis MW (2019) Using DeepLabCut for 3D markerless pose estimation across species and behaviors. Nat Protoc 14:2152–2176. 10.1038/s41596-019-0176-0 31227823

[B36] Nodal FR, Bajo VM, Parsons CH, Schnupp JW, King AJ (2008) Sound localization behavior in ferrets: comparison of acoustic orientation and approach-to-target responses. Neuroscience 154:397–408. 10.1016/j.neuroscience.2007.12.022 18281159 PMC7116516

[B37] Parker PRL, Abe ETT, Leonard ESP, Martins DM, Niell C (2022) Joint coding of visual input and eye/head position in V1 of freely moving mice. Neuron 110:3897–3906. 10.1016/j.neuron.2022.08.029 36137549 PMC9742335

[B38] Pelli DG (1997) The VideoToolbox software for visual psychophysics: transforming numbers into movies. Spat Vis 10:437–442. 10.1163/156856897X00366 9176953

[B39] Pollard JS, Beale IL, Lysons AM, Preston AC (1967) Visual discrimination in the ferret. Percept Mot Skills 24:279–282. 10.2466/pms.1967.24.1.279

[B40] Prusky GT, Alam NM (2013) Behavioral measurement of mouse visual function. In: *Behavioral genetics of the mouse* (Crusio WE, Sluyter F, Gerlai RT, Pietropaolo S, eds), pp 45–54. Cambridge, UK: Cambridge University Press.

[B41] Schall JD (2001) Neural basis of deciding, choosing, and acting. Nat Rev Neurosci 2:33–42. 10.1038/35049054 11253357

[B42] Shepherd SV, Platt ML (2006) Noninvasive telemetric gaze tracking in freely moving socially housed prosimian primates. Methods 38:185–194. 10.1016/j.ymeth.2005.12.003 16431130 PMC1592521

[B43] Singh VP, Li J, Dawson K, Mitchell JF, Miller CT (2025) Active vision in freely moving marmosets using head-mounted eye tracking. Proc Natl Acad Sci U S A 122:1–12. 10.1073/pnas.2412954122 PMC11831172 39899712

[B44] Skyberg RJ, Niell CM (2024) Natural visual behavior and active sensing in the mouse. Curr Opin Neurobiol 86:1–9. 10.1016/j.conb.2024.102882 PMC11254345 38704868

[B45] Spering M (2022) Eye movements as a window into decision-making. Ann Rev Vis Sci 8:427–448. 10.1146/annurev-vision-100720-125029 35676097

[B46] Stitt I, Zhou ZC, Radtke-Schuller S, Frohlich F (2018) Arousal dependent modulation of thalamo-cortical functional interaction. Nat Commun 9:1–14. 10.1038/s41467-018-04785-6 29941957 PMC6018110

[B47] Sugrue LP, Corrado GS, Newsome WT (2005) Choosing a greater of two goods: neural currencies for valuation and decision making. Nat Rev Neurosci 6:363–375. 10.1038/nrn1666 15832198

[B48] von Melchner L, Pallas SL, Sur M (2000) Visual behavior mediated by retinal projections directed tot he auditory pathway. Nature 404:871–876. 10.1038/35009102 10786793

[B49] Wallace DJ, Greenberg DS, Sawinski J, Rulla S, Notaro G, Kerr JND (2013) Rats maintain an overhead binocular field at the expense of constant fusion. Nature 498:65–69. 10.1038/nature12153 23708965

[B50] Wallace DJ, et al. (2025) Eye saccades align optic flow with retinal specializations during object pursuit in freely moving ferrets. Curr Biol 35:1–15. 10.1016/j.cub.2024.12.03239909033

[B51] Wispinski NJ, Gallivan JP, Chapman CS (2020) Models, movements, and minds: bridging the gap between decision making and action. Ann N Y Acad Sci 1464:30–51. 10.1111/nyas.13973 30312476

[B52] Zangemeister WH, Stark L (1982) Types of gaze movement: variable interactions of eye and head movements. Exp Neurol 77:563–577. 10.1016/0014-4886(82)90228-X 7117463

[B53] Zoccolan D, Graham BJ, Cox DD (2010) A self-calibrating, camera-based eye tracker for the recording of rodent eye movements. Front Neurosci 4:1–12. 10.3389/fnins.2010.00193 21152259 PMC2998901

