# Supplementary figures and images for: Heading and Then Saccades Predict Visual Discrimination Decisions in Freely Moving Ferrets
Source: eNeuro. 2026 May 20;13(5):ENEURO.0124-26.2026. doi: 10.1523/ENEURO.0124-26.2026 (PMC13197153; doi:10.1523/ENEURO.0124-26.2026)

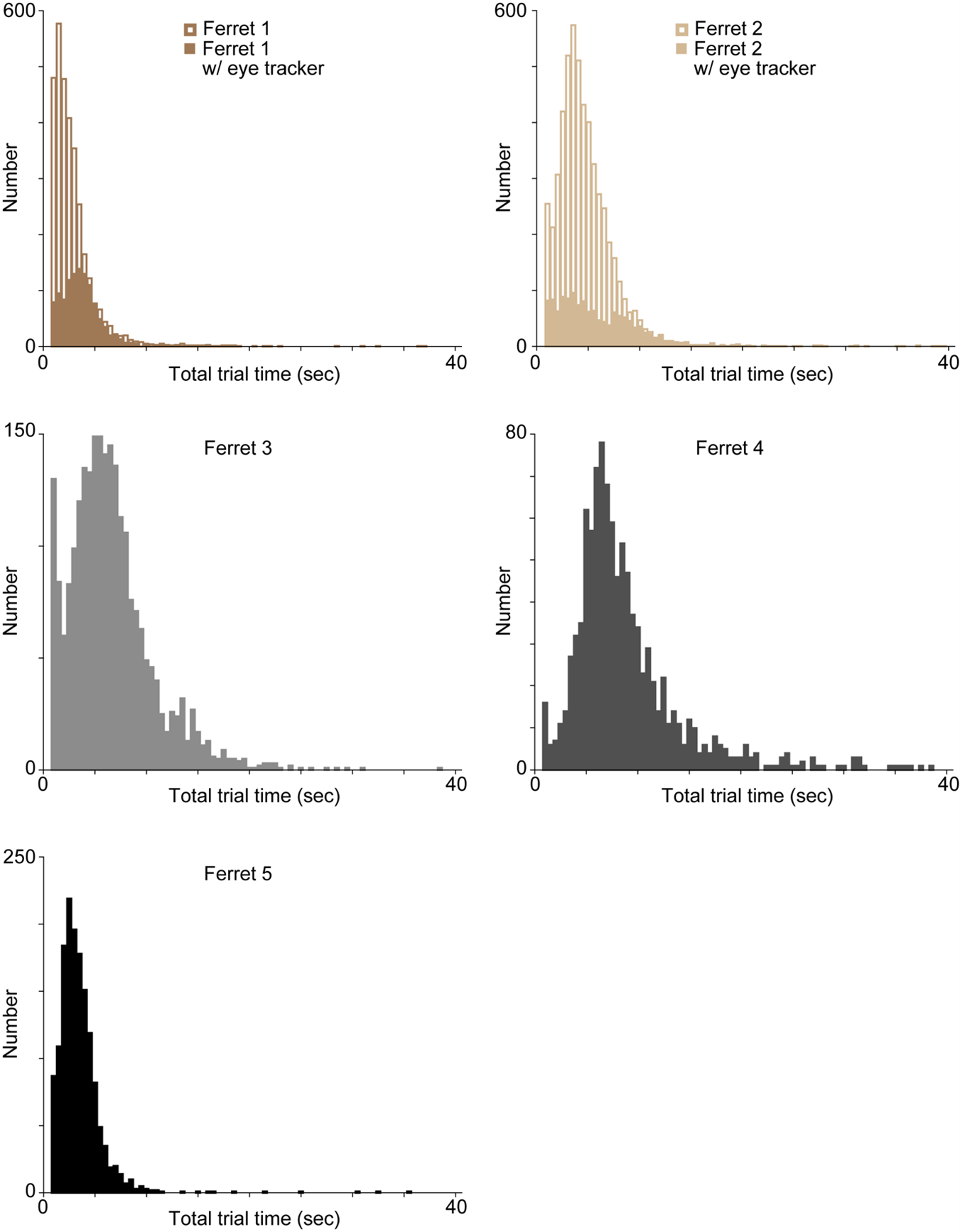

Supplement: Figure 1-1 — Distributions of total trial times for each ferret. For Ferrets 1 and 2 (top row), data from sessions with and without the eye tracker are color-coded according to the legend. Download Figure 1-1, TIF file. [file eneuro-13-ENEURO.0124-26.2026-s007.tif]

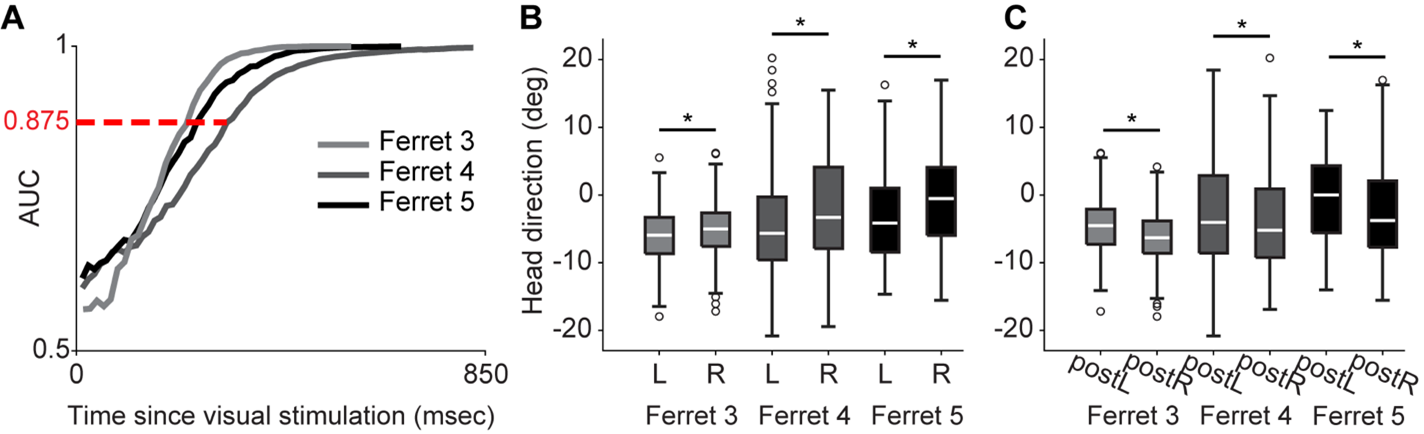

Supplement: Figure 2-1 — Head tracking indicates decision time, turning time, and decision bias in Ferrets 3, 4, and 5 (no eye tracker). A. Area under the ROC curves (AUC) of head direction predictive of correct choice calculated from all trials for 3 additional ferrets. AUC of 0.875 (red dashed line) is the decision criterion from which decision time was assessed (see Methods). B. Distributions of head direction at stimulus onset prior to left (L) or right (R) choice per ferret. White lines are medians, the bottom and top edges of the boxes indicate the 25th and 75th percentiles, respectively, whiskers indicate the range of data not considered outliers, and circles are outlier data points. Asterisks indicate significant differences between eventual left/right choices (see Table 3 for statistics). C. Distributions of head direction at stimulus onset following previous trial left (L) or right (R) choice per ferret. Conventions as in B, see Table 3 for statistics. Download Figure 2-1, TIF file. [file eneuro-13-ENEURO.0124-26.2026-s008.tif]

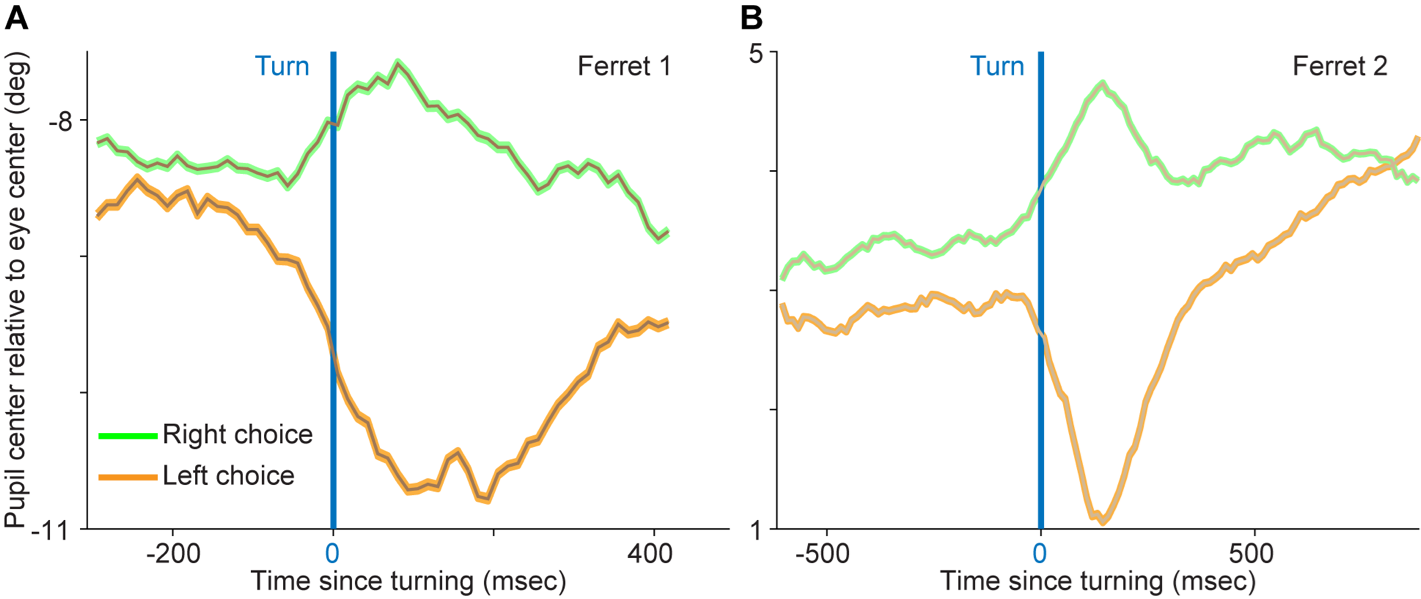

Supplement: Figure 3-1 — Eye position relative to turning time. Mean eye position (degrees in horizontal direction, relative to the center of the eye) aligned to the turning time (blue line) per trial in Ferret 1 (A) and Ferret 2 (B). Green and orange curves show data prior to right and left choice, respectively. Shaded areas show SEMs. Note differences in timescales across ferrets. Download Figure 3-1, TIF file. [file eneuro-13-ENEURO.0124-26.2026-s009.tif]

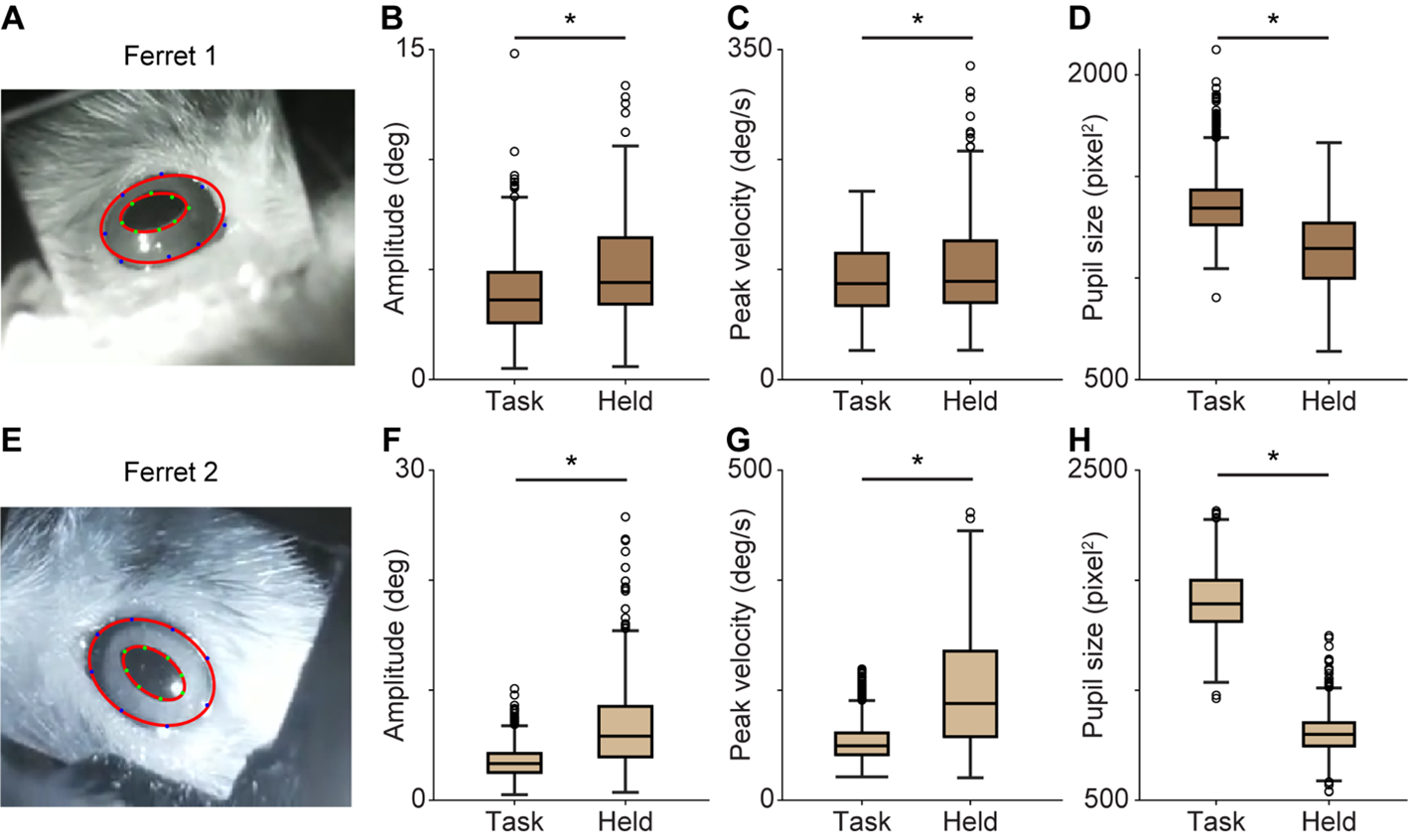

Supplement: Figure 4-1 — Eye tracking in Ferrets 1 and 2 while hand-held. A. Example video frame of eye tracking in Ferret 1. The boundaries of the pupil and the eyelid were labeled automatically using DeepLabCut and fit with ellipses (red). B-D. Saccade amplitude (B), peak velocity (C), and pupil size (D) in Ferret 1 when performing visual discrimination tasks compared to being held. Black lines are medians, the bottom and top edges of the boxes indicate the 25th and 75th percentiles, respectively, whiskers indicate the range of data not considered outliers, and circles are outlier data points. Asterisks indicate significant differences across conditions (see Figure 4-2 for statistics). E-H. Pupil tracking and saccade and pupil size data for Ferret 2, conventions as in A-D. Download Figure 4-1, TIF file. [file eneuro-13-ENEURO.0124-26.2026-s010.tif]
