# Supplementary material for: Heading and Then Saccades Predict Visual Discrimination Decisions in Freely Moving Ferrets
Source: eNeuro. 2026 May 20;13(5):ENEURO.0124-26.2026. doi: 10.1523/ENEURO.0124-26.2026 (PMC13197153; doi:10.1523/ENEURO.0124-26.2026)
Supplement: Figure 4-2 — Saccades. Saccade amplitude (degrees in any direction), peak velocity (deg/sec in any direction), and pupil size (mean ± SD) were measured while ferrets performed a task or were hand-held. N’s are total number of trials. P values (unpaired t-tests) were calculated for comparisons of each metric across the two conditions. Bold P values indicate significant differences. Download Figure 4-2, DOCX file. [file eneuro-13-ENEURO.0124-26.2026-s011.docx]

**Figure 4-2**

**Figure 4-2. Saccades.** Saccade amplitude (degrees in any direction), peak velocity (deg/sec in any direction), and pupil size (mean ± SD) were measured while ferrets performed a task or were hand-held. N’s are total number of trials. P values (unpaired t-tests) were calculated for comparisons of each metric across the two conditions. Bold P values indicate significant differences.

|  |  | **Amplitude (deg)** | **Peak velocity (deg/sec)** | **Pupil Size (pixel^2^)** |
| --- | --- | --- | --- | --- |
| **Ferret 1** | Task (n = 904) | 3.8 ± 1.8 | 108.3 ± 38.8 | 1363.3 ± 152.3 |
|  | Held (n = 441) | 4.9 ± 2.3 | 117.8 ± 50.4 | 1139.5 ± 193.7 |
|  | P value | **5.3E-23** | **1.5E-4** | **2.0E-99** |
| **Ferret 2** | Task (n = 1547) | 3.5 ± 1.4 | 90.0 ± 30.5 | 1709.8 ± 188.4 |
|  | Held (n = 1033) | 6.6 ± 3.7 | 166.1 ± 85.2 | 906.3 ± 115.2 |
|  | P value | **1.0E-170** | **1.2E-191** | **0 (extremely small)** |
