# Supplementary material for: Heading and Then Saccades Predict Visual Discrimination Decisions in Freely Moving Ferrets
Source: eNeuro. 2026 May 20;13(5):ENEURO.0124-26.2026. doi: 10.1523/ENEURO.0124-26.2026 (PMC13197153; doi:10.1523/ENEURO.0124-26.2026)
Supplement: Figure 5-1 — Largest saccades. Horizontal distance (mean ± SD) in degrees, negative indicates leftward and positive indicates rightward, of the largest amplitude saccades in left- or right-choice trials. N’s are total number of trials. Download Figure 5-1, DOCX file. [file eneuro-13-ENEURO.0124-26.2026-s012.docx]

**Figure 5-1**

**Figure 5-1**. **Largest saccades**. Horizontal distance (mean ± SD) in degrees, negative indicates leftward and positive indicates rightward, of the largest amplitude saccades in left- or right-choice trials. N’s are total number of trials.

|  | **Ferret 1 w/ eye tracker** | **Ferret 2 w/ eye tracker** |
| --- | --- | --- |
| **Left choice** | -2.8 ± 3.8 (n = 206) | -3.0 ± 3.3 (n = 271) |
| **Right choice** | 3.0 ± 3.5 (n = 231) | 2.9 ± 2.7 (n = 237) |
